# Improvement of Phosphorus Use Efficiency in Rice by Adopting Image-Based Phenotyping and Tolerant Indices

**DOI:** 10.3389/fpls.2021.717107

**Published:** 2021-08-31

**Authors:** Bishal Binaya Bhatta, Rajendra Kumar Panda, Annamalai Anandan, Nirakar Susanta Narayan Pradhan, Anumalla Mahender, Kumbha Karna Rout, Bhaskar Chandra Patra, Jauhar Ali

**Affiliations:** ^1^Crop Improvement Division, Indian Council of Agricultural Research-National Rice Research Institute, Cuttack, India; ^2^Department of Plant Physiology, Orissa University of Agriculture and Technology, Bhubaneswar, India; ^3^Rice Breeding Innovation Platform, International Rice Research Institute (IRRI), Los Baños, Philippines

**Keywords:** rice, phosphorus use efficiency, image J, geometric traits, breeding

## Abstract

Phosphorus is one of the second most important nutrients for plant growth and development, and its importance has been realised from its role in various chains of reactions leading to better crop dynamics accompanied by optimum yield. However, the injudicious use of phosphorus (P) and non-renewability across the globe severely limit the agricultural production of crops, such as rice. The development of P-efficient cultivar can be achieved by screening genotypes either by destructive or non-destructive approaches. Exploring image-based phenotyping (shoot and root) and tolerant indices in conjunction under low P conditions was the first report, the epicentre of this study. Eighteen genotypes were selected for hydroponic study from the soil-based screening of 68 genotypes to identify the traits through non-destructive (geometric traits by imaging) and destructive (morphology and physiology) techniques. Geometric traits such as minimum enclosing circle, convex hull, and calliper length show promising responses, in addition to morphological and physiological traits. In 28-day-old seedlings, leaves positioned from third to fifth played a crucial role in P mobilisation to different plant parts and maintained plant architecture under P deficient conditions. Besides, a reduction in leaf angle adjustment due to a decline in leaf biomass was observed. Concomitantly, these geometric traits facilitate the evaluation of low P-tolerant rice cultivars at an earlier stage, accompanying several stress indices. Out of which, Mean Productivity Index, Mean Relative Performance, and Relative Efficiency index utilising image-based traits displayed better responses in identifying tolerant genotypes under low P conditions. This study signifies the importance of image-based phenotyping techniques to identify potential donors and improve P use efficiency in modern rice breeding programs.

## Introduction

Phosphorus, a macronutrient placed in phosphorus (P) block, maintains overall plant morphometry, regulates metabolic processes, and is a fundamental element of several essential biomolecules (nucleic acid, ATP, NADPH, and phospholipids) involved in reproduction, pointing to its indispensability in crop growth and development. However, its less solubility and very low concentration in the soil rhizosphere of about 0.05–0.3 μg P ml^−1^ (Bolan, 1991) severely limit crop yield. The phosphorus use efficiency (PUE) of rice is only about 25% (Dobermann and Frairhurst, 2000), and it absorbs 1.07 M tonnes of P_2_O_5_ at the rate of 24.3 kg P_2_O_5_ ha^−1^ alone, providing enormous scope for its development. Meanwhile, the report suggests that India demands 19% of the global production of phosphate fertiliser (Tirado and Allsopp, 2012), bagging its limelight. The chelation of P with Fe and Al in acidic; Ca in alkaline soil and immobilised in fine textured clay-loam soil intensifies P deficiency. Moreover, statistics shows that 20 million hectares of world rice cultivation area are Pi-deficient (Neue et al., 1990), and that 61.02% of Indian soil is low in P (Muralidharudu et al., 2011). To alleviate this issue, the idea of developing P use-efficient rice cultivars has been introduced as an efficient strategy, especially in India, to reduce the cost of production, import demand, and effect of eutrophication (Mahender et al., 2017).

In rice, screening strategies for the identification of genotypes with low P tolerance or improved P acquisition ability the plant need to be harvested or destroyed at certain stages and evaluated based on several parameters, such as relative tillering ability (RTA), shoot and root biomass production, and root architectural response, such as root hair, axial root angle, elongation of lateral roots with high specific root length value under low P conditions (Zhu and Lynch, 2004; Vandamme et al., 2013). Among these traits, those of root is the critical aspect for improving crop performance *via* improving P acquisition (Richardson et al., 2011; Rose et al., 2013a,b). However, observing root traits is a labour-intensive, time-consuming process that only includes selective traits. It provides inaccurate data as it depends on means of subsets of plants at each harvest rather than follows the growth trajectories of individuals (Berger et al., 2013). To overcome this difficulty, the advent of proximal sensing technology allows for critical non-destructive support in measuring performance and predicting crop yield under controlled and field environments (Araus and Cairns, 2014; Araus and Kefauver, 2018). A non-invasive technique, such as high-throughput image-based phenotyping, e.g., visible imaging or RGB (red-green-blue) imaging, has a potential application, as it screens the varieties at earlier stages, covering crucial parameters such as vegetative mass associated with important traits such as grain yield in cereals (Ghamkhar et al., 2019; Anandan et al., 2020). It allows the measurement of dynamic changes in the plant form over time and quantifies based on multiple parameters such as tillering, leaf area index, leaf angle, and convex hull (Anandan et al., 2020).

Consequently, the imaging technique is gaining momentum among researchers to screen genotypes for several biotic and abiotic factors such as salinity, nitrogen, water deficiency, nodal root angle, and early seedling vigour in barley, rice, and sorghum (Araus et al., 2012; Crowell et al., 2014; Atkinson et al., 2015; Turner et al., 2018; Narisetti et al., 2019; Wu et al., 2019; Anandan et al., 2020). However, the phenotypic screening of rice genotypes under low phosphorus conditions is not established. In this study, we followed a systematic phenotypic strategy of destructive and non-destructive image-based phenotyping methodologies applied to examine the complex of genotype × phenotype × environment interactions in a low phosphorus regime at an early stage, which, to the knowledge of the authors, is the very first case study. Initially, to understand the plant response to low P using manual morphometric traits, 65 popular improved rice varieties were screened, and selected rice genotypes were evaluated to identify the traits through destructive (morphology and physiology) and non-destructive (geometric characteristics by imaging) techniques. The screening methodologies in both experiments were helpful in identifying the tolerance genotypes, and used the derivative index traits for low P tolerance, which is demonstrated here. This approach is most suitable for low P tolerance and improving the PUE in rice breeding programs. Based on this rationale, this experiment was set to (i) differentiate rice genotypes by exploring all possible physiological and geometric (non-destructive) traits by multivariate analysis, (ii) identify geometric characteristics by imaging and with associated morphological traits to use them as a surrogate in the absence of an imaging system, and (iii) demonstrate different adaptive mechanisms involved in low P tolerance.

## Materials and Methods

### Plant Materials

Sixty-five popular rice genotypes developed for Odisha province and three checks (Dular, Kasalath, and IC459373) (Supplementary Table 1) were collected from Regional Research and Technology Transfer Station (RRTTS), Coastal zone, Bhubaneswar; Orissa University of Agriculture and Technology (OUAT); and ICAR-National Rice Research Institute (NRRI), Cuttack, Odisha.

### Experiment-1: Soil-Based Screening

This experiment was conducted using a cemented tank (1 m × 10.29 m × 2.3 m) at NRRI, Cuttack (20°27'09” N, 85°55'57” E, 26 msl), Odisha, containing low P soil (<3 kg/ha; 0–15 cm layer, pH 4.9) collected from Central Farm, OUAT, Bhubaneswar, Odisha. Before sowing, the seeds of all the genotypes were heat-treated to break seed dormancy in a hot-air oven at 50°C for 45 h. The experiment was conducted in January 2019 for 45 days (day/night temperature of 23.5/16°C, RH ~70%, bright sunlight) without the addition of any fertiliser. Seeds of the 68 genotypes were directly sown 15 cm apart in a 1-m long row with a spacing of 20 cm between rows in a randomised block design with three replications. The uniform plant population of two seedlings per hill was maintained across genotypes by thinning on the 14th day after sowing, and irrigated on alternate days until the end of the experiment. For post-uprooting of plants, on the 45th day, morphometry traits such as shoot length (cm), maximum root length (cm), number of tillers per plant, root dry weight (g), shoot dry weight (g), percent dry matter partitioning for root, and root: shoot ratio (based on length) were taken into consideration for the identification of genotypes and for the subsequent hydroponic experiment.

### Experiment-2: Hydroponic Study

From experiment 1, 18 genotypes (Supplementary Table 1) were selected based on the level of tolerance from all quarters of principal component analysis (PCA) to evaluate further under hydroponics and identify the traits through destructive (morphology and physiology) and non-destructive (geometric traits by imaging) techniques in a low phosphorus regime. Uniform size seeds of respective genotypes were handpicked carefully to avoid admixture and heat-treated in the hot air oven at 50°C for 45 h to break the seed dormancy. Subsequently, surface sterilisation with 75% ethanol for 1 min and 2.5% sodium hypochlorite for 20 min (NaClO) was performed. To remove the traces of the sterilising agent, the seeds were washed four to five times in sterile distilled water for several minutes. Two seeds of each genotype were placed on Styrofoam fixed with mesh. The Styrofoam containing seeds was placed in a plastic tray containing 10 L of tap water for 3 days in the dark to promote germination. Later, the Styrofoam with well-germinated and healthy seedlings was transferred to a tray containing full strength of Yoshida solution (10 L/tub) (Yoshida et al., 1976) as control. Parallel to the control, another set of genotypes was placed in Yoshida solution containing 0.5 ppm (deficient P) of NaH_2_PO_4_.H_2_O. The experiment was performed with three replications for 35 days. The pH of the nutrient solution was adjusted between 4.5 and 4.55 on an alternate day, and tap water was added to compensate for the loss in the solution. Once every 7 days, a new solution was used with respective concentrations by maintaining the same pH.

### Sample Collection and Data Observation by Destructive and Non-destructive Technique

On the penultimate day of the experiment, SPAD (SPAD-502, Konica Minolta, Tokyo, Japan) value was observed on the fourth leaf from the bottom to measure chlorophyll concentration. The plants were separated from Styrofoam carefully to record the plant images 36 days after sowing (DAS). Photographs of three plants per genotype of three biological replicates were taken, and 10 plants were used to measure the morphometric data to estimate growth parameters on the same day. The images were captured using a 12-megapixel Nikon camera (Nikon, Tokyo, Japan) at 1.5 m with high precaution. The plants were imaged under a high-intensity artificial light source inside a closed chamber. To calculate the whole plant area (WPA), a ruler was placed near the plants with proper labelling. The images were recorded from three different angles; front and backside views (at 90°) and top view, which fall in the visible range or the so-called RGB spectrum (400–700 nm). Uniformity was maintained in all aspects, such as light intensity and distance from plants, throughout the process.

The recorded images were analysed for geometric traits using the open-source Image J software. Briefly, excess areas on all four sides of the selected images were cropped, and only potted plants were composed of one object. A straight line with known distance was drawn over the ruler for further image analysis. The plant images were then separated from the background with the colour threshold system. To measure individual or whole plant area, the greenness of the plant was determined by converting RGB images into Hue Saturation and Brightness (HSB) system. Using a known scale, the individual or whole plant area was interchanged from pixels to mm^2^. The summed area of all three images (top and two side views) was used to estimate the whole plant area (WPA) and expressed in square millimetres.

**Figure 1:**
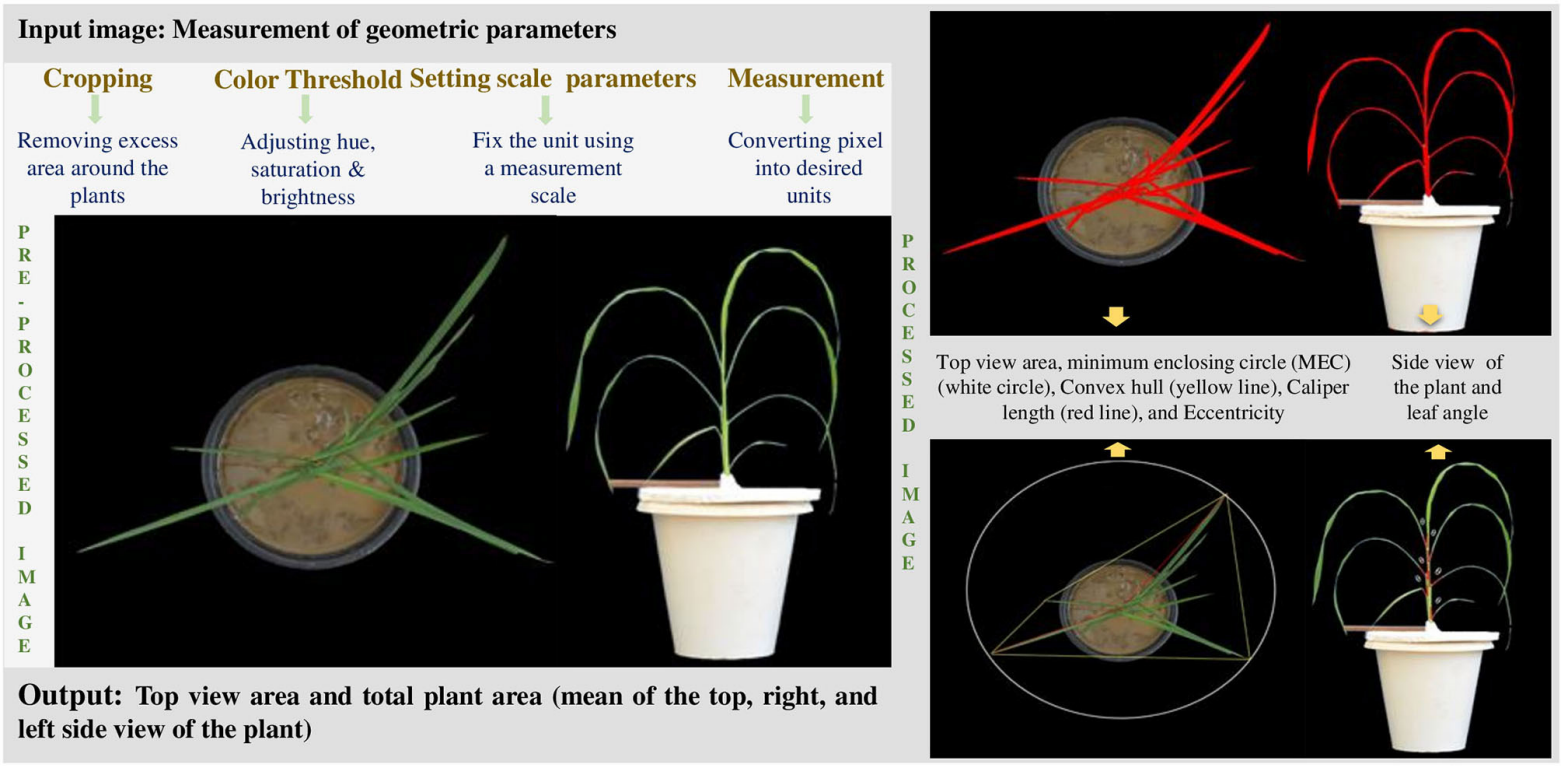
Image processing and derivation of geometric parameters with representative plant image.

Additionally, other geometric traits such as convex hull (the smallest possible mathematically solved perimeter that envelopes the imaged plant), calliper length (the longest dimension of the canopy when viewed from above), minimum enclosing circle (MEC) (the minimum circle that can enclose the plant), and eccentricity (the degree of radial symmetry) were measured from images of the top view (Neilson et al., 2015; Anandan et al., 2020), while leaf inclination at all levels (adaxial side) was analysed using Image J from the image taken from the front view (Figure 1). To measure the leaf inclination, the stem was considered as the vertical axis. The leaf blade inclined from the lamina joint junction was noted, and the angle was drawn between the two points (leaf blade and lamina joint) to determine the angle (Figure 1).

The same plants and additional seven plants per replication of each genotype were used to observe morphological traits such as shoot length (cm), maximum root length (cm), number of roots, number of leaves plant^−1^, number of tillers^−1^, shoot dry weight (g), stem dry weight (g), root dry weight (g), and individual leaf weight (g) at respective positions. The first formed leaf from base was counted as the first leaf, and successive leaves were numbered accordingly. Besides, root traits such as total root length (cm), projected root area (cm^2^), root surface area (cm^2^), average root diameter (mm), root volume (cm3), and the number of root tips, were determined from three plants per biological replicate using WinRHIZO^TM^ (Régent Instruments Inc., 2013, Quebec City, Canada).

The dry weight of shoot, root, stem, and leaves was taken after drying the detached part in a hot air oven at 60°C for 5 to 6 days until the samples were dried completely. Thereafter, the dried samples were finely ground, and around 300 mg shoot and 90 mg root samples were used for total P quantification following the ternary acid method. To quantify P from the shoot and root, samples were taken in a digestion tube and kept in the digestion unit for 1:45 to 2:15 h at 150 to 170°C for digestion in the ternary acid mixture (conc. HNO_3_ + conc. H_2_SO_4_ + conc. HClO_4_: 5:1:2). The digest P concentration was determined using Systronics (Gujarat, India) UV Spectrophotometer at 420 nm, and total shoot and root P contents were determined on an mg/g dry weight basis. Phosphorus utilisation efficiency of the shoot (PUE_S) and phosphorus utilisation efficiency of root (PUE_R) were calculated using the following formula.

$$\begin{array}{l} \text{Phosphorus utilization efficiency }\left(\text{PUE}\right)\;=\\ \;\frac{\left(\text{P content in control }-\text{ P content in deficient conditions}\right)}{\text{Phosphorus applied}} \end{array}$$

### Statistical Analysis

Principal component analysis was performed with 68 rice genotypes for seven traits to identify the genotypes based on their performance under low P conditions. Initially, the mean data of all the traits were calculated. The PCA analysis was executed using the PCA function from the Facto Mine R package (Lê et al., 2008) in R version (3.6.3) (R Core Team, 2015). Among the 68 genotypes, 18 representative genotypes were selected based on their response under low P conditions and were further analysed under hydroponic conditions.

Descriptive statistics, ANOVA, variability and heritability analyses were performed for both control and P deficiency conditions for the selected 18 genotypes evaluated under hydroponic conditions to assess the effect of P-induced changes under deficient conditions using PBTools v1.4 (PBTools, 2014). The model used for ANOVA was

$$\begin{array}{l} Yijk=\mu +\alpha i+\beta j+\gamma ij+\varepsilon ijk \end{array}$$

where μ is the overall mean, α*i* is the effect of the *i*th genotype, β*j* is the effect of the *j*th P concentration, γ*ij* is the effect due to any interaction between the ith genotype, jith P is the concentration, and ε*ijk* is the error. The genotypes and P levels were considered as fixed, while replicates and interactions were considered as random.

Broad sense heritability (H^2^), for each trait in different concentrations of P, was estimated as

$$\begin{array}{l} H^{2}=\sigma^{2}g/\sigma^{2}p\;\times \;100\;and\;\sigma^{2}p=\sigma^{2}g+\sigma^{2}e/r \end{array}$$

where σ^2^g = genotypic variances, σ^2^p = phenotypic variances, σ^2^e is the error variance, and r is the number of replications.

The variability in traits related to phosphorus deficiency tolerance was studied by principal component analysis on a matrix of 38 morphometric and geometric traits of the 18 genotypes. To examine the effect of low P on the different genotypes, the Pearson correlation among all the morphological and geometric traits was analysed using the corrplot functions from the corrplot package (Wei et al., 2017) in R. The clustering approach was executed separately for the genotypes and traits studied under P deficiency conditions. The output of the clustering technique would be useful to identify genotypes having similar phenotypic expression and traits that are similar under P deficiency conditions. To carry out the heatmap analysis, genotypes of both treatments are arranged in rows and traits in columns. The cluster heat map function (x, dendrogram = “both,” scale = “row,” method = “ward.D^2^”) of the R package made4 (Culhane et al., 2005) was used to identify the possible tolerance mechanisms for P deficiency using the 18 genotypes (both treatments) and 38 traits. In keeping with the rest of the functions, dendrogram = “none” was used to avoid phylogeny, and to understand the differential trait expression between treatments.” Row Z-score scaling method was used to normalise the data of each row (trait). Z = (x – μ)/σ, where x is the trait value, μ is the population means of the trait, and σ is the population standard deviation. The colour spectrum (legend) illustrates the Z-score across the range of −2 to 2. A Z-score of ‘0’ indicates that the trait value is identical to the mean, and values greater than the mean are categorised into +2 and vice versa.

### Contribution of Individual Plant Parts Towards Its Overall Development

It explains the nature of the contribution of the individual plant part, especially leaf in orchestrating plant growth and development, calculated using the following formulas:

- Contribution of the individual leaf for its own development = (individual leaf weight/total leaf weight) *100.
- Contribution of the individual leaf towards total development = (individual leaf dry weight/total dry weight) *100.
- Percentage partitioning of dry matter = (individual plant part/total dry weight) *100.
- Contribution of the leaf towards shoot development = (individual leaf dry weight/shoot dry weight) *100.
- Contribution of the leaf towards root development = (individual leaf dry weight/root dry weight) *100.

### Tolerance Indices

Several researchers have proposed screening indices to determine the level of tolerance among genotypes evaluated in controlled and stress environments (Table 1). These stress indices may be used as an indicator to identify tolerant genotypes that perform well under P deficiency conditions. In this study, the secondary trait that generates maximum variability from the above-mentioned statistical analysis was substituted in the indices to distinguish genotypes that perform well under deficient conditions.

**Table 1:** Tolerance and susceptibility indices formula used in this study.

**Index**	**Formula**	**Selection pattern**	**References**
Mean Productivity Index (MPI)	MPI = (Y_i_) ns + (Y_i_) s/2	Maximum value	Hossain et al., 1999
Mean Relative Performance (MRP)	[(Y_i_) s/(Ys)] + [(Y_i_) ns/(Yns)]	Maximum value	–
Relative Efficiency Index (REI)	[(Y_i_) s/(Ys)] * [(Yi) ns/(Yns)]	Maximum value	–
Stress Tolerance Level (TOL)	TOL = (Y_i_) ns - (Y_i_) s	Minimum value	Rosielle and Hamblin, 1981
Stress Tolerance Index (STI)	[(Y_i_) ns*(Y_i_) s]/(Yns)^2^	Maximum value	Fernandez, 1992
Stress Susceptibility Index (SSI)	SSI = [1– (Y_i_) s/(Y_i_) ns]/SI.	Minimum value	Fischer and Maurer, 1978
Drought Tolerant Efficiency (DTE)	(Specific trait under stress/Specific trait under non-stress)* 100	Maximum value	Fischer and Wood, 1981

*(Y_i_) ns, specific trait of ith genotype under non-stress conditions; (Y_i_) s, specific trait of ith genotype under stress conditions; SI (stress intensity), 1– (Ys/Yns); Ys, mean of the specific trait of all the genotypes evaluated under stress conditions; Yns, mean of the specific trait of all the genotypes evaluated under non-stress conditions*.

## Results

### Experiment 1

#### Multivariate Analysis to Assess Low P-Tolerant Rice Genotypes in P-Deficient Soil

To generate a core set of low P tolerant genotypes for experiment 2, the growth and performance of 68 genotypes (Supplementary Table 1) were studied in P-deficient soil by PCA considering eight morpho-physiological parameters. Principal component 1 (PC1) and PC2 individually accounted for 53.6 and 20.9% of total variability among traits and individuals, respectively, representing a cumulative variance of 74.5%. PCA revealed that total biomass showed the highest degree of positive correlation to PC1, followed by root and shoot biomass. Similarly, root: shoot ratio (based on length) contributes maximally to PC2 followed by dry matter partitioning for root. Genotypes plotted on the right side of PCA of origin were found to exhibit better tolerance, as all the traits clustered together, showing maximum variation to these two components (Supplementary Figure 1). Conversely, genotypes belonging to –PC1 and +PC2 are sensitive, as no other traits possess variability. Based on the output of the PCA, 18 genotypes were selected from all quarters based on the superiority or inferiority of the traits such as root length, shoot dry weight, root dry weight, and shoot length. Traits such as root length (mean = 11.63 cm) shoot dry weight (mean = 0.157 g), root dry weight (mean = 0.066 g), and shoot length (mean = 22.46 cm) that exhibited maximum contribution in the PCA analysis were selected to identify genotypes for hydroponic experiment. The mean of the respective traits was considered as a threshold in the selection of the genotypes.

### Experiment 2

#### Degree of Variation in Physio-Morphological Traits Under P Deficient and Sufficient Conditions

Morphological, physiological, and geometric traits of the 18 rice genotypes were studied under P sufficient (control) and deficient conditions. Analysis of variance reflected significant differences among the genotypes, P concentrations (C), and their interaction (G × C) for most of the studied parameters (Table 2). Among the morphological and physiological traits, shoot length, leaf number, maximum root length, number of roots, SPAD, dry biomass of third to seventh leaf angle, stem, shoot and root, shoot and root P content, and their utilisation efficiency significantly (*P* < 0.01) differed between genotypes under deficient, control, and interaction conditions. The geometric trait, convex hull, root volume, and root tips significantly differed at both genotype and P concentration, while, calliper length, minimum enclosing circle (MEC), and eccentricity had a significant difference only at the genotype level. However, tiller number, whole plant area (WPA), top view area (TVA), and third to seventh leaf angles were strongly influenced by P concentration.

**Table 2:** Analysis of variance for various traits under deficiency (0.5 ppm) and control concentration of phosphorus and genotype interaction effect.

**Variate**	**Genotype (G)** **MSS (0.5 ppm)**	**Genotype (G)** **MSS (control)**	**Genotype (G)** **MSS of interaction**	**G SS%**	**Conc. MSS**	**Con. SS%**	**G x C MSS**	**G × C** **SS%**
Shoot length	<0.01	<0.01	<0.01	85.95	<0.01	5.41	<0.05	4.48
Tiller number	ns	ns	ns	15.54	<0.01	25.11	ns	16.26
Leaf number	<0.01	<0.01	<0.01	36.11	<0.01	41.77	<0.01	12.18
Root number	<0.01	<0.01	<0.01	65.34	<0.01	15.44	<0.01	11.76
Root length	<0.01	<0.01	<0.01	59.35	<0.01	19.76	<0.05	9.92
SPAD	<0.01	<0.01	<0.01	53.61	<0.05	2.34	<0.05	23.85
1st leaf weight	ns	ns	ns	31.06	ns	3.64	ns	29.93
2nd leaf weight	ns	<0.01	<0.01	61.65	<0.05	4.60	ns	9.45
3rd leaf weight	<0.01	<0.01	<0.01	62.07	ns	0.04	<0.01	20.94
4th leaf weight	<0.01	<0.01	<0.01	79.73	ns	0.25	<0.05	10.30
5th leaf weight	<0.01	<0.01	<0.01	78.44	<0.01	2.63	ns	7.32
6th leaf weight	<0.01	<0.01	<0.01	27.91	<0.01	38.76	<0.01	20.78
Stem dry weight	<0.01	<0.01	<0.01	80.67	<0.01	3.82	<0.01	10.26
Shoot weight	<0.01	<0.01	<0.01	59.13	<0.01	20.24	<0.01	11.86
Root dry weight	<0.01	<0.01	<0.01	70.55	<0.01	15.31	<0.01	8.33
Whole plant area	<0.05	ns	ns	19.26	<0.01	32.32	ns	16.48
Top view area	ns	<0.05	<0.05	26.33	<0.01	27.17	<0.05	21.88
Shoot P	<0.01	<0.01	<0.01	2.17	<0.01	96.08	<0.01	1.47
Root P	<0.05	<0.01	<0.01	4.37	<0.01	90.37	<0.01	3.65
Convex hull	<0.01	<0.05	<0.01	57.05	<0.01	11.69	ns	10.19
Calliper length	<0.01	ns	<0.01	55.31	ns	0.14	ns	16.21
Eccentricity	<0.01	<0.05	<0.01	70.22	ns	0.05	ns	6.94
Mini enclosing circle	<0.01	<0.05	<0.01	61.98	ns	1.40	<0.05	16.54
1st Leaf angle	ns	<0.01	ns	28.46	ns	1.29	ns	27.74
2nd Leaf angle	ns	<0.01	<0.01	40.18	ns	0.24	<0.05	29.10
3rd Leaf angle	ns	ns	<0.01	37.59	<0.01	16.95	ns	16.83
4th Leaf angle	ns	ns	<0.01	28.73	<0.01	36.90	ns	10.24
5th Leaf angle	ns	ns	ns	16.23	<0.01	33.49	ns	21.76
6th Leaf angle	ns	ns	ns	17.60	<0.01	28.27	ns	18.26
T root Length	ns	<0.01	<0.01	45.46	<0.01	10.93	<0.01	22.82
Proj. root Area	ns	<0.01	<0.01	55.72	ns	0.53	<0.05	22.72
Root Surf. Area	ns	<0.01	<0.01	55.72	ns	0.53	<0.05	22.72
Root avg diameter	<0.01	ns	<0.01	38.90	<0.01	20.91	ns	16.70
Root volume	<0.05	<0.01	<0.01	57.29	<0.05	2.52	ns	18.12
Tips	<0.05	<0.01	<0.01	35.11	<0.01	15.78	ns	26.44
PUE_S	<0.01	<0.01	<0.01	2.52	<0.01	95.16	<0.01	1.71
PUE_R	<0.01	<0.01	<0.01	5.06	<0.01	88.24	<0.01	4.28

*P-values shown as “ns” (non-significance) >0.05. PUE_S, phosphorus utilisation efficiency of shoot; PUE_R, phosphorus utilisation efficiency of root*.

The results revealed that traits other than leaf angle, genotype, and P concentration significantly influence most of the traits in comparison with G × C interaction. All these outcomes suggest that the genetic basis of the trait under study can be exploitable for a further breeding program. The G × C interaction provides deeper insight into the adaptability of genotypes in a deficient environment. Among the traits studied, genotype explained most variation (28–86%), while traits, namely, tiller number, leaf number, sixth, and seventh leaf weight, WPA, TVA, shoot and root P, fourth to sixth leaf angle, and PUE of shoot and root were explained by P concentration.

Descriptive statistics for 38 traits of the 18 genotypes under control and low P conditions are shown in Supplementary Table 2. Under deficient conditions, stem dry weight exhibited the highest (92%) degree of heritability followed by root dry weight, shoot length, fourth leaf weight, shoot P content, shoot PUE, and root length exhibited more than 80% (Supplementary Table 2). The image-based geometric traits such as MEC (77%) and convex hull (73%) showed better heritability responses than the other geometric traits. More than 60% of the traits were normally distributed with absolute values of skewness of <1. The coefficient of variation (CV) was high for most of the studied parameters, 3 to 64% under low P conditions, and 6 to 78% under control conditions (Supplementary Tables 2, 3).

**Figure 2:**
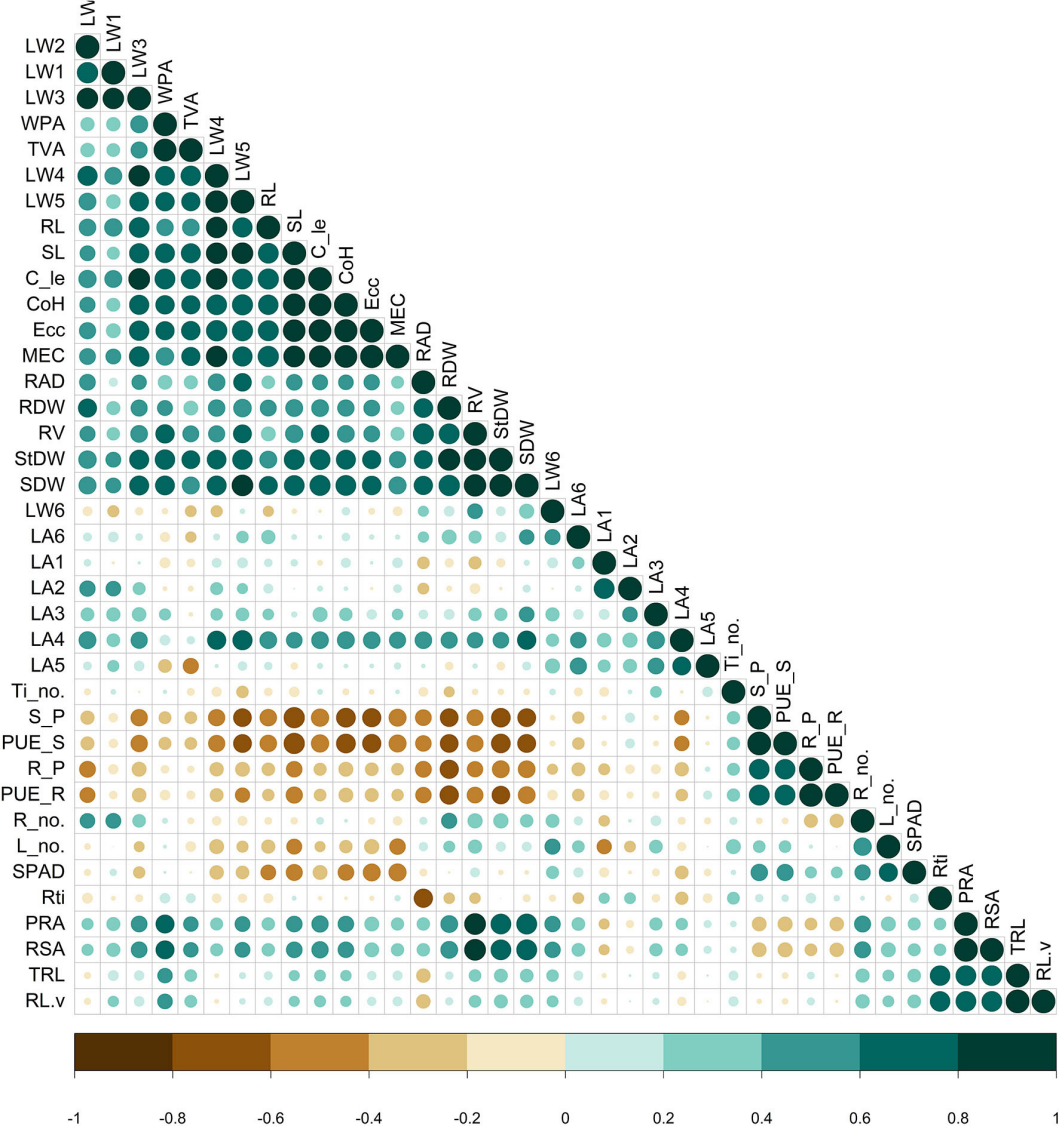
Pearson correlation matrix of the measured traits in the phosphorus (P) stress environment. Colour (green-positive correlation; brown-negative correlation) intensity and the size of circle are proportional to the correlation coefficient. RAD, average root diameter; RDW, root dry weight; RV, root volume; StDW, stem dry weight; SDW, shoot dry weight; LW, leaf weight; WPA, whole plant area; TVA, top view area; RL, maximum root length; SL, shoot length; C_le, calliper length; CoH, convex hull; Ecc, eccentricity; MEC, minimum enclosing circle; LA, leaf angle; Ti_no, tiller number; S-P- shoot P content; PUE_S, PUE of the shoot; R_P, root P content; PUE_R, PUE of the root, Rti, root tip; TRL, total root length; PRA, projected root area; RSA, root surface area; R_no.- number of roots, L_no., leaf number.

**Figure 3:**
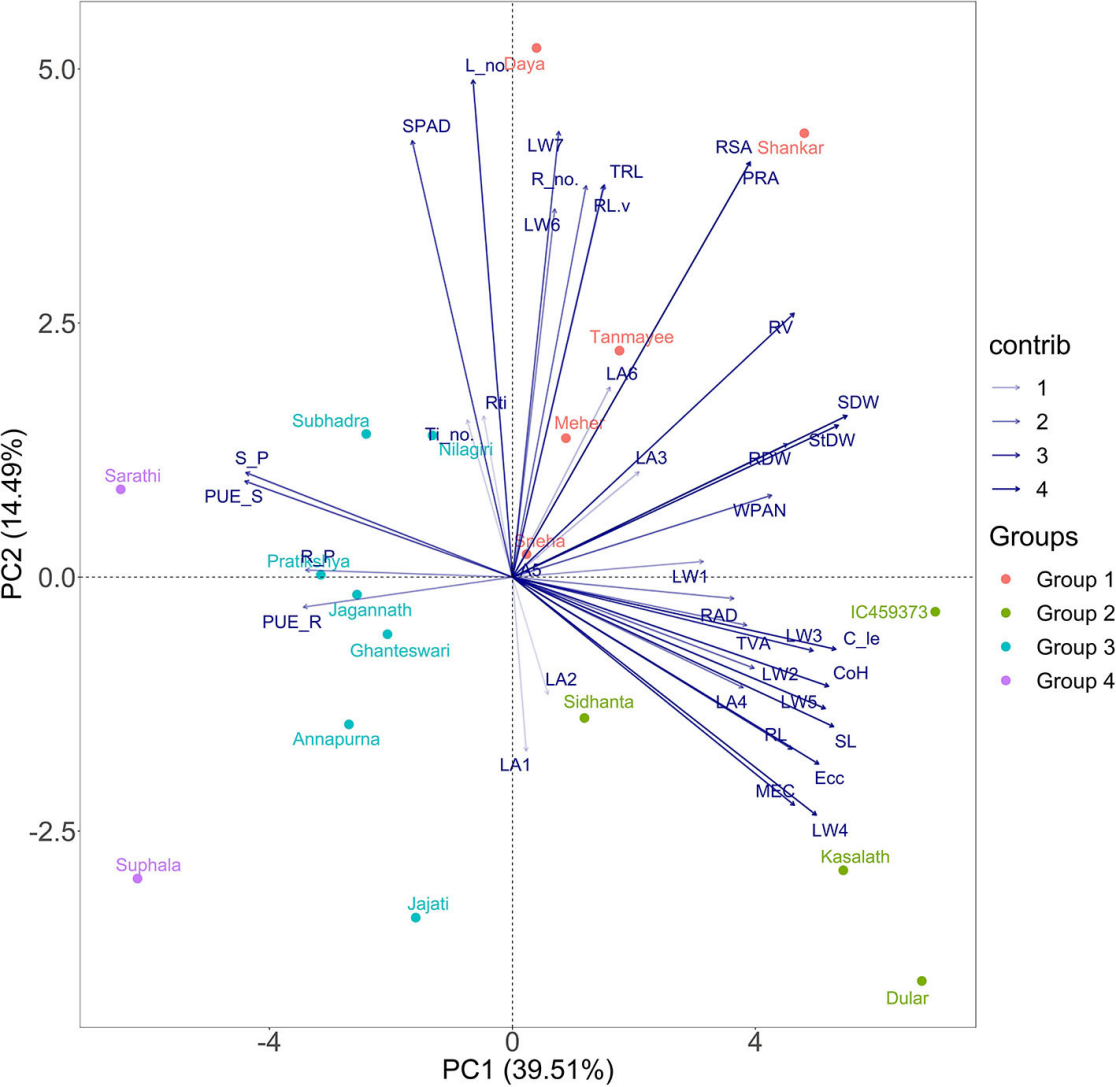
Principal component analysis (PCA) biplot of the 18 genotypes based on variance in 38 morpho-physiological and geometric traits measured in P stress environment, explained by two axes. Together, the two PC axes explained 54% of the total variance. The transparency of the vector indicates the contribution to the variance in the dataset, ranging from 1 (lightest) to 4% (darkest). The direction and length of the vector represent trait contribution to the first two components of the PCA. Genotypes are grouped into four based on their expression pattern of morpho-physiological and geometric traits measured under deficient P conditions. Group 1 (orange circle) shares high values of root surface area, Group 2 (green circle) shares high values of leaf area and weight, and Group 3 (cyan colour) and Group 4 (purple colour) share high values of the shoot and root P content with reduced growth.

The correlation matrix (Figure 2) displayed traits with significance under P deficient conditions. Among the geometric traits, the MEC, convex hull, calliper length, and eccentricity showed a significant strong positive correlation (>0.90, *P* < 0.01) between them. Similarly, they had a strong positive association with TVA, WPA, third, fourth, and fifth leaf weight, shoot length, stem dry weight, shoot dry weight, root volume, and root length, which is in support of the PCA-derived results (Figure 3). For the physiological traits, root P content showed a significant positive association with root PUE (0.9731, *P* < 0.01). However, shoot PUE and shoot P content displayed a greater negative association with the convex hull (−0.6829, *P* < 0.05; −0.6828, *P* < 0.05) and eccentricity (−0.6419, *P* < 0.05; −0.6418, *P* < 0.05). Similarly, shoot length is significantly negatively correlated with shoot PUE (−0.7887, *P* < 0.05) and shoot P content (−0.7886, *P* < 0.05). Coherently, root surface area and root volume positively correlated with shoot weight (0.7662) and fifth leaf weight (0.626, *P* < 0.05). Interestingly, the average root diameter exhibited a strong positive association with root volume, shoot, stem, and root biomass, and negatively correlated with the number of root tips (−0.6532, *P* < 0.05). Physiological traits such as individual leaf weight also manifested positive responses among each other. The dry weight of all leaves at each level had positively correlated with the preceding two leaves.

#### Genotype and Trait Grouping Reveals the Significance of Traits Under Deficient Conditions

Hierarchical clustering classified the 18 genotypes into two major clusters, *viz*., cluster 1 (12 genotypes) and cluster 2 (6 genotypes), differing in their level of expression of morpho-physiological and geometric parameters under deficient P conditions (Supplementary Figure 2). Each sub-cluster serially from first to three comprises four (sub-cluster 1), five (sub-cluster 2), and three (sub-cluster 3) genotypes, respectively. Among the three sub-clusters, sub-cluster 3 exhibited maximum values for all geometric, morphological, and physiological traits, namely, leaf inclination except root number, SPAD, total root length, root tips, project, and surface area of roots. Interestingly, sub-cluster 1 displayed a bit lower values in all the above-mentioned traits of sub-cluster 3 with maximum total root length, root tips, project, and surface area of roots. On the other hand, the contribution of all the traits declined in sub-cluster 2 when compared with sub-clusters 3 and 1. Contrastingly, cluster 2 displayed lower contributions from almost all the traits except for root (0.64) and shoot P (0.66) contents.

Clustering of genotypes reflected the significance of the geometric traits as well as the morpho-physiological traits, which are again reflected from trait clusters based on Ward's D distance (Supplementary Figure 3). It grouped all the traits into six clusters. MEC and convex hull formed individual clusters, terming them as the most valuable non-destructive predictors of low P tolerance under deficient conditions. Similarly, clusters 3 and 4 encompassed 30 and 4 traits, respectively. Additionally, WPA and TVA form cluster 5 separately showed their significance under P deficient conditions. Traits in each cluster exhibit more closeness compared with those of the other clusters, which are again throw-back by the tree form of trait cluster representation.

#### PCA Revealed the Tendency of Genotypes and Contributions of Traits Under P-Deficient Conditions

Principal component analysis was performed to determine the principal components of morpho-physiological and geometric parameters of the 18 rice genotypes, which best explains the response to low P conditions for screening tolerant rice varieties under hydroponic study. The first two principal component vectors contribute 39.51 (PC1) and 14.49% (PC2) to the total variation, which collectively describes a cumulative variance of 54% (Figure 3). From the selected traits, shoot dry weight followed by stem dry weight exhibited a very strong positive correlation to dimension 1. Among the geometric traits, which are new to this study, convex hull and calliper length showed promising results compared with the other geometric traits. In contrast, root and shoot P contents, and root and shoot PUEs illustrate a negative correlation on PC1. Similarly, morphological traits such as leaf numbers have a strong correlation followed by SPAD for dimension 2. However, root traits such as root number, surface and projected root area, total root length, and root volume contributed more towards dimension 2. Dimension 3 explained 9.93% of the total variation. The number of root tips preceded by TRL possessed strong interrelations among the traits.

As PC1 and PC2 contributed more than half of the total variation and held significant importance in separating the genotypes into several categories, the 18 rice genotypes were classified into four distinct groups, similar to hierarchical clustering. The genotypes present at the third quadrant exhibited a maximum level of expression of geometric traits having the highest degree of tolerance in the low P regime, which are IC459373, Dular, Kasalath, and Sidhanta. Similarly, genotypes such as Shankar, Meher, Tanmayee, Sneha, and Daya at the second quadrant, depending upon their position in the ordinal plane and direction, exhibited maximum root growth with the moderate geometric trait. Genotypes Jajati, Annapurna, Ghanteswari, Jagannath, Pratikshya, Nilagiri, and Subhadra were grouped as moderately sensitive based on their response under low P conditions. On the left plane of the plot, Suphala and Sarathi fell at the end of –PC1 and +PC2, forming the most sensitive ones.

#### Variation in Low P Tolerance Exists Across Landraces and Improved Rice Genotype Mechanism

The performance of the 18 rice genotypes was further scrutinised with a clustered heat map approach under both control and low P conditions. Albeit this approach is complex, it is more informative. It not only provides information on essential traits but also visualises the responses of genotypes in different colour shades. Hierarchical clustering separates the genotypes into two major clusters, which are further divided into several sub-clusters. In major cluster 1, Dular, Kasalath, IC459373, and Sidhanta performed better under deficient conditions, grouping them as tolerant ones, and were clustered with genotypes (Sidhanta, Shankar, Tanmayee, Jajati, and Meher) that performed well under control conditions. The majority of the traits, such as leaf weight, root dry weight, stem dry weight, and average root diameter at all levels, and geometric traits such as MEC and Convex hull positively attributed to this cluster and followed the same grouping pattern as was evident from the trait cluster. Major cluster 2 is represented by two sub-clusters as deficient and control groups. In sub-cluster 1, genotypes (Sneha, Subhadra, Nilagiri, Suphala, Pratikshya, Sarathi, and Jagannath) poor in their performance under low P conditions were grouped. Sub-cluster 2 is a group of a mixture of several genotypes, and included those that were moderately tolerant to low P. Among them, Shankar and Tanmayee illustrated two contrasting scenarios where the former was in a better position than the latter under deficient conditions, making Shankar more tolerant than Tanmayee. On the other hand, genotypes Jagannath and Suphala underperformed under both deficient and control conditions, indicating their sensitivity and making them more susceptible than the other genotypes. Therefore, this approach provides a better look at variation among genotypes under different conditions.

#### Contribution of Individual Plant Parts Towards Its Overall Development

The results of this study shed light on the physiological aspect of plant development. Leaves, the major photosynthetic organ of plants, allocate assimilates to different plant parts and contribute towards its overall development. Fifth leaf (31.44%) followed by the fourth leaf (22.96%) and sixth leaf (16.01%) from the base contribute photosynthates maximally for its development across all the genotypes under P deficient conditions. Similarly, the fifth leaf (9.02%) followed by the fourth leaf (7.06%) exhibited a greater assimilated contribution towards overall plant development. Moreover, under low P conditions, the greater dry matter that was diverted to stem (15.94%) followed by root (10.48%) indicated that maintenance of above-ground parts in a deficient environment is more important than that of below-ground parts for balancing yield. Additionally, the fifth leaf (20.14, 89.22%) preceded by the fourth leaf (15.78, 69.47%) contributed maximally towards both shoot and root development, irrespective of all genotypes displaying their importance.

#### Variation in Root Diameter and Number of Root Tips Across Various Rice Genotypes

Both root diameter and number of root tips are key components of the underground plant part, mainly associated with nutrient sensing and its uptake. The results depicted the percentage variation in average root diameter and number of root tips across various rice genotypes under limited P conditions. Most of the genotypes exhibited variation in average root diameter that ranged from 5.78 to 55.77% (Supplementary Table 4). This suggests that there is an increase in root diameter under low P conditions over the control environment. However, some cultivars such as Annapurna (−3.63%) and Ghanteswari (−3.26%) displayed a reverse trend. Nilagiri showed the highest positive variation in average root diameter (55.77%) followed by Dular (55.18%), Kasalath (39.07%), and IC459373 (35.74%). However, most of the cultivars showed negative variation in the number of root tips under stress conditions compared with the control that ranged between −2.25 and −65.81%. Several genotypes reported positive changes, such as Sarathi (38.72%), Ghanteswari (13.49%), Daya (3.72%), and IC459373 (32.08%). However, genotypes such as Dular (−65.81%) suggested the highest negative variation followed by Shankar (−63.04%), Pratikshya (−50.29%), Meher (−43.9%), Nilagiri (−40.01%), Jagannath (−28.93%), Tanmayee (−20.22%), and Suphala (−6.94%).

#### Comparison of Specific Traits Based on Stress Indices

Several stress indices had been developed to be used as an indicator for identifying tolerant genotypes evaluated in abiotic stress environments. Based on the contribution of the specific trait, the stress indices were grouped into two categories; tolerant indices (MPI, MRP, REI, STI, and DTE) and susceptible indices (TOL and SSI). The correlation between the indices and various traits considered, MPI, MRP, and REI, had a strong correlation that ranged between 0.646 and 0.988 (Table 3). Among the various traits, shoot length, displayed a greater degree of positive correlation with MPI (*r* = 0.982, *P* < 0.01), MRP (*r* = 0.984, *P* < 0.01), and REI (*r* = 0.988, *P* < 0.01) followed by stem dry weight and 5th leaf weight. Out of various geometric traits, MEC revealed a better positive relationship with MPI (*r* = 0.953, *P* < 0.01), MRP (*r* = 0.96, P <0.01), and REI (*r* = 0.962, *P* < 0.01) followed by the convex hull and calliper length. Besides, other traits such as shoot dry weight manifested positively with the above-described tolerance indices (MPI, MRP, and REI) followed by average root diameter, fourth leaf weight, root dry weight, third leaf weight, fourth leaf angle, total root length, and number of root tips.

**Table 3:** Relationship (*r*) between tolerance indices and some selected traits measured under P deficient conditions.

**Trait**	**TOL**	**STI**	**SSI**	**DTE%**	**MPI**	**MRP**	**REI**
Shoot length	0.161	0.056	−0.056	0.056	0.982	0.984	0.988
Stem dry weight	0.389	0.024	−0.024	0.024	0.971	0.978	0.97
5th leaf weight	−0.074	0.397	−0.397	0.397	0.973	0.973	0.983
Mini. enclosing circle	0.243	0.27	−0.27	0.27	0.953	0.96	0.962
Convex hull	−0.05	0.61	−0.61	0.61	0.932	0.953	0.918
Avg. root diameter	−0.911	0.838	0.838	0.838	0.963	0.952	0.952
Shoot dry weight	0.424	−0.191	0.191	−0.191	0.923	0.938	0.945
4th leaf weight	−0.2	0.175	−0.175	0.175	0.936	0.938	0.936
Calliper length	0.183	0.021	−0.021	0.021	0.934	0.935	0.938
Root dry weight	−0.511	0.037	0.037	0.037	0.957	0.918	0.919
SPAD	−0.787	0.792	−0.792	0.792	0.911	0.915	0.913
4th Leaf angle	0.042	0.345	−0.345	0.345	0.803	0.907	0.94
3rd leaf weight	−0.491	0.549	0.549	0.549	0.862	0.861	0.851
Leaf number	−0.177	0.311	−0.311	0.311	0.825	0.846	0.839
3rd Leaf angle	−0.263	0.41	−0.41	0.41	0.764	0.839	0.881
Total root length	−0.203	0.334	−0.334	0.334	0.72	0.759	0.741
Root tips	−0.475	0.57	−0.57	0.57	0.646	0.734	0.745
Top view area	−0.074	0.294	−0.294	0.294	0.417	0.622	0.608
5th Leaf angle	−0.568	0.76	−0.76	0.76	0.197	0.619	0.657
Whole plant area	0.021	0.11	−0.11	0.11	0.381	0.599	0.585

## Discussion

Nutrients and their uptake and/or utilisation efficiency are the most evergreen debatable topics haunting the scientific community. Since P, an element with immense importance, not only nurtures all biological species but also forms the basis of life, including the plant community. Exasperated with the conventional method, non-invasive RGB imaging has been included to identify the tolerant plant in early stages encompassing crucial geometric traits, such as leaf angle, MEC, and leaf area, associated with grain yield. To unfold the traits related to low P, conventional and geometric traits measured by imaging technique were applied for the 18 genotypes identified from initial soil-based screening selected from 65 genotypes by applying multivariate analysis, and mean of root length, shoot length, and biomass of shoot and root were considered as a threshold (Supplementary Figure 1). In this study, we have established the possibility of using non-destructive imaging techniques to differentiate genotypes at an early stage of crop growth in a P-deficient environment, identification of the most informative traits indicating the tolerant source, and their potential underlying mechanisms for low P tolerance.

The analysis of variance suggests leaf angle as an adaptive trait, as it is greatly influenced by P concentration and unravels geometric traits such as calliper length, MEC, and eccentricity significantly differed at the genotype level. Among the variance, genotype (G) has a maximum contribution followed by concentration (C) and G × C interaction. However, traits, namely, number of tillers, WPA, TVA, and leaf angle of position from third to seventh were immensely influenced by P availability, hinting at their sensitivity towards it. The trait stem dry weight exhibits the highest heritability at a low P concentration, which is the most important quantitative parameter considered in the evaluation of low P-tolerant crop plants. However, physiological traits stem and root dry weight, fourth leaf weight, shoot P content, and its utilisation efficiency exhibited higher heritability in both P regimes, possibly because of constitutive gene expression in a particular tissue. Additionally, most of the traits displayed a greater degree of variation, as it is evident from their CV and controlled genetically, clearly showing from GCV values (Supplementary Tables 2, 3). Therefore, the higher CV specifies exploitation of a higher degree of genetic variability among the studied parameters and the possibility of a greater potential in selecting these parameters in developing low P-tolerant genotypes.

To assess the performance of the genotypes under phosphorus-deficient conditions, several traits were reported, such as shoot and root biomass, tiller number, total root length, volume, and phosphorus content of shoot on the destructive basis (Rose et al., 2016; Anandan et al., 2021). In this study, we found that the traits such as shoot and root biomass, stem weight, root length, root volume, and P content of shoot were highly correlated and significant with low P tolerance. Therefore, we have focussed the attention on these representative traits, and the geometric traits measured using an imaging technique have become the epicentre of this study. The geometric trait calliper length positively correlated with third, fourth, and fifth leaf weights (Figure 2). Similarly, the convex hull displayed a highly significant correlation with calliper length and with the weight of third, fourth, and fifth leaves. The stronger association between them suggests greater biomass accumulation, full expansion of leaves, and wider convex hull under P-deficient conditions clearly supports that these traits would play an essential role in differentiating lines. Fully expanded active leaves absorb a high amount of solar radiation, have a high CO_2_ assimilation rate, and translocate large amounts of assimilates to other parts of the plant. Furthermore, MEC also positively correlated with third, fourth, and fifth leaf weights, calliper length, convex hull, eccentricity, shoot length, and root length, indicating dependency of those traits influencing yield. The top view area and WPA were positively correlated with the leaf weight of third to fifth levels and fourth leaf angle along with the above-described geometric traits (calliper length, convex hull, eccentricity, and MEC) and morphological trait shoot length. This indicates that leaves positioned at 3rd to 5th perform better nutrient mobilisation among different parts of plants by maximising leaf area when viewed from the top. Furthermore, the fully expanded leaves with a good number of tillers maintain higher ground coverage by foliage, thus reducing the weed population by facilitating rapid vegetative growth under low P conditions. The trait WPA was positively associated with calliper length, convex hull, eccentricity, fourth and fifth leaf weights, combining root architectural traits such as root volume, projected area, and surface area. This suggests that increased root area and volume increases WPA because of greater excavation of nutrients either by diffusion and/or by ion exchange by contact with nutrients through enhancement in root traits (Reddy et al., 2020). This analysis revealed that genometric traits (MEC, convex hull, calliper length, eccentricity, etc.) and morphological traits (shoot length, shoot and root dry weight, total root length, root surface area, root volume, root diameter, projected root area, etc.) have been further utilised as predictors for low P tolerance under P-deficient conditions. From a physiological point of view, the fifth leaf followed by the fourth and sixth leaves supported their growth maximally, indicating them to be the most active photosynthetic organs, but, due to the senescence of the first leaf and very young growth of the seventh leaf, did not contribute much to its development. Similarly, the fifth and fourth leaves also maximally contributed to overall plant shoot and root development, indicating these to be the most active photosynthetic reservoir. Greater dry matter diversion towards shoot rather than root implies the importance of shoot architecture in determining final crop yield. While considering the root-associated traits, maximum root length and root volume were found to have a prime role under P deficient conditions. They exhibited the strongest association (*r* = > 0.7, *P* < 0.05) with most of the traits (Figure 2). Both of them (root length and volume) had no negative association with the other traits but had a moderate association (*r* = 0.4) between them, having some traits in common with a positive association. Therefore, by improving root length and volume together, there is a possibility to explore greater soil volume to enhance P uptake from a distant region of soil under P-deficient conditions. On the contrary, the increase in average root diameter had a negative effect on the number of root tips, P content, and PUE of shoot and root. The average root diameter was found to increase with a decrease in the availability of P (Anandan et al., 2021). Compared with control, root diameter was increased by 18.91%, and root tips decreased by 25.46% at 0.5 ppm. The decrease in the number of root tips might be due to an increase in root diameter with aerenchyma, root length (maximum and total root length), and restricted branching. This could be a strategy to reduce the energy requirement for root growth and maintenance (Steffens and Rasmussen, 2016) under P-deficient conditions.

In this experiment, the testing of different genotypes revealed that root dry weight showed a negative correlation with root and shoot phosphorus contents, and utilisation efficiency, hinting that the observed P was not available in sufficient quantity under phosphorus-deficient conditions. Plants with large roots have the opportunity to explore more nutrients, but that does not necessarily mean higher P uptake, which is clear from the negative correlation between root dry weight and root or shoot P content. Further, the negative association between root length and shoot P or shoot utilisation efficiency supports poor uptake or poor translocation of P from root to shoot. Similarly, a negative association was observed for shoot length, and shoot and stem biomass (Figure 2). Among the various geometric and physiological traits, the negative association of shoot P content and its utilisation efficiency with leaf weight (third to fifth), convex hull, MEC, calliper length, and eccentricity explained why those traits need more P than any of the other traits to maintain the plant in required architecture. Presumably, those traits may compensate the P deficit through remobilization from fully and partly senescenced leaves that might be acting as organic P pools.

**Figure 4:**
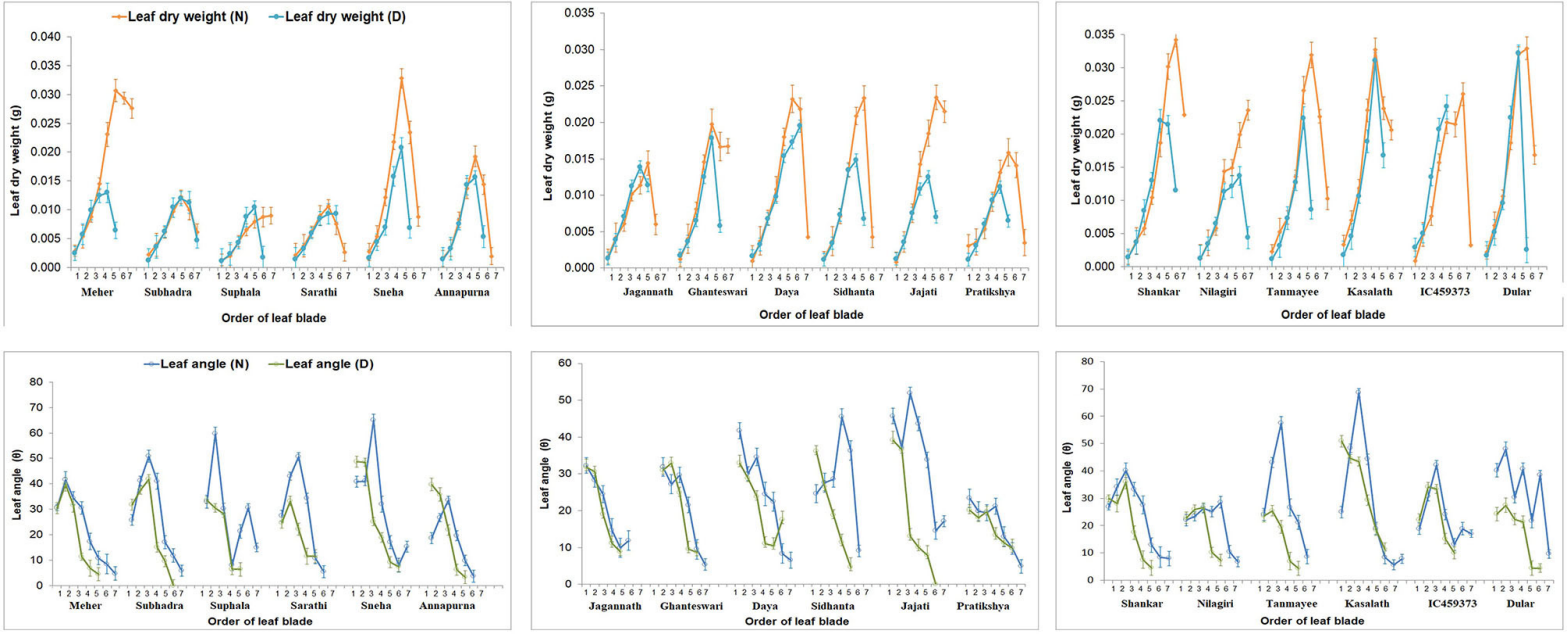
Effect of P on leaf blade weight and leaf angle of the 18 genotypes at all levels and relative comparison between the treatments. Individual points with error bars refer to leaf position from bottom to top. Leaf blade weight and leaf angle were reduced at all levels of leaves. Tolerant genotypes exhibited minimum relative differences for leaf blade and angle.

The hierarchical clustering that separated MEC, and convex hull into two separate clusters from the rest of the other traits suggests their importance in differentiating the genotypes under deficient conditions compared with other traditionally (morphological and physiological) measured traits. The two geometric traits (MEC and convex hull) that form separate clusters depend on the development and inclination of fully functional leaves. They are directly related to the area, biomass accumulation, and inclination of leaves under both control and deficient P conditions. Widely, all cultivars maintained higher leaf biomass under control than deficient conditions (0.085 ± 0.034). Interestingly, susceptible genotypes Subhadra, Sarathi, Jagannath, and Suphala had minimum differences in leaf biomass at all levels under limited P conditions with high tissue P (>0.7 mg/g) and PUE (>0.0036) (Figures 3, 4). This could be the dilution effect. The smaller leaves appeared to have higher P concentration compared with genotypes having larger leaves. Besides, these susceptible genotypes exhibited the least TVA (<1,600 mm^2^) under P-deficient conditions. High tissue P, and the PUE of smaller and narrow leaves would be a strategy of plants to improve their survival under P-deficient conditions. Further research on these genotypes would help gain insight into understanding the minimum differences in leaf biomass between control and deficient conditions.

**Figure 5:**
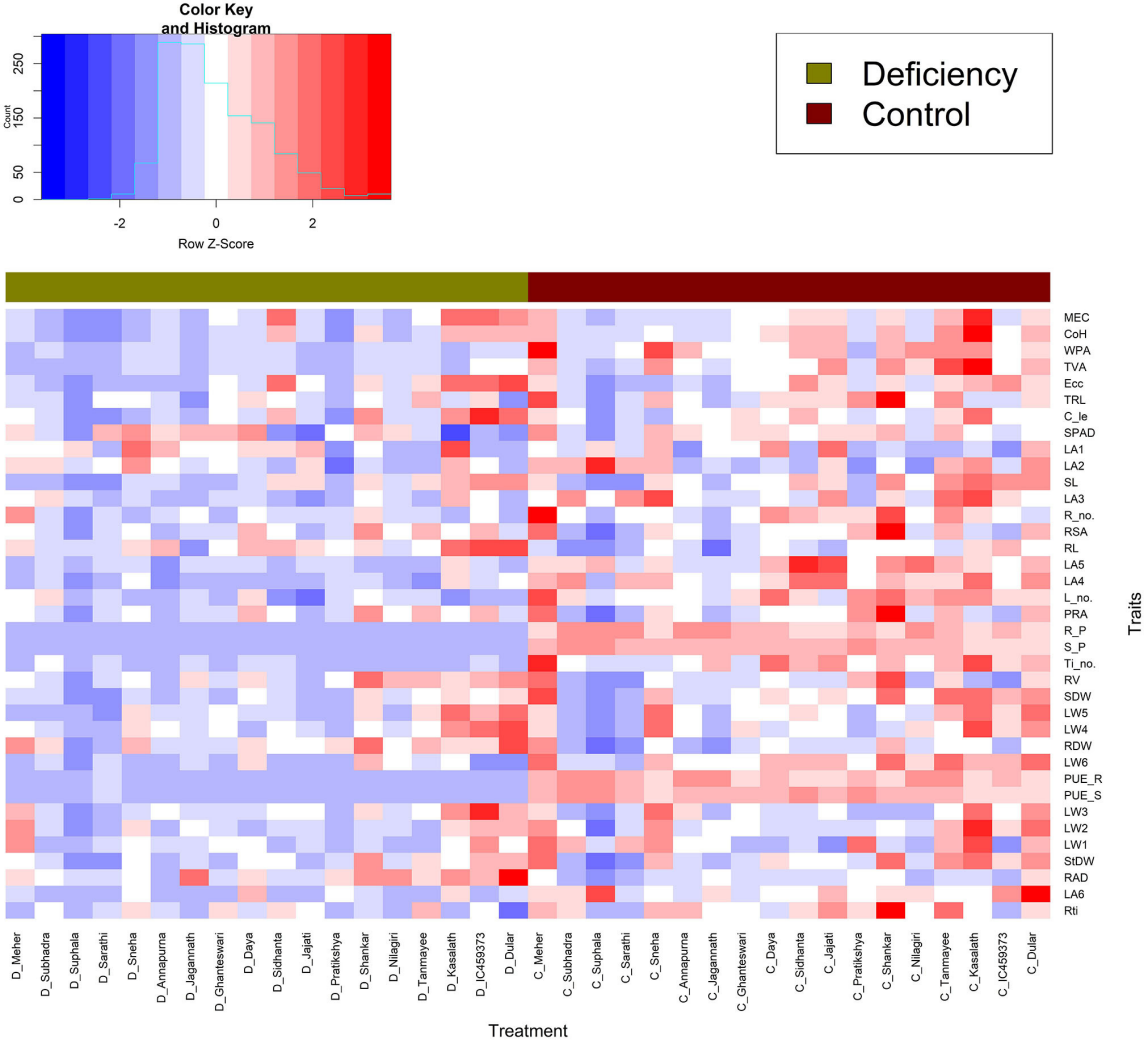
Heat map of two treatments (control and deficit) of the 18 genotypes and 38 morpho-physiological and geometric traits. Each column represents a genotype, and each row represents a trait. The horizontal bar with two colours (olive and maroon) represents the treatment difference.

The close relationship between leaf dry weight and angle summarises that more biomass accumulation in leaves would lead to a higher rate of leaf inclination and vice versa. This can be well-observed from the positive association between them (Figure 4). However, the negative correlation between first leaf weight and angle might be because the first formed leaf (primary leaf) is small and acts as a protective covering for the succeeding leaf (Dunand and Saichuk, 2014), and that it is associated with initial root growth (Anandan et al., 2020). On the other hand, Anandan et al. (2021) reported reduced leaf area and increased root growth in 2-week-old rice seedlings under P-deficient conditions. Apart from that, a reduction in leaf inclination might be due to the adaptiveness of rice plants under P-deficient conditions. P-deficient plants reduce photosynthesis to conserve Pi. Plants raised under low P conditions inhibit the export of triose-P from chloroplast stroma to the cytosol by the Pi translocators (Natr, 1992), leading to the conversion of photosynthate into starch in the chloroplast. The Pi that liberated on the conversion of triose-P into starch and consumed in large amounts during photosynthesis for the synthesis of ATP was stopped to conserve the use of Pi (Dietz and Foyer, 1986). This highlights that the geometric traits MEC and convex hull, based on leaf inclination, would involve manifesting their importance as non-destructive parameters to differentiate the genotypes in a low P environment. Additionally, other traits such as total root length, root surface area, root volume, projected root area, shoot P content, and utilisation efficiency establish their vitality in the selection of low P-tolerant rice genotypes. The heat map in Figure 5 gives an enhanced picture of traits expression between the two groups (control and deficit). Traits such as an increase in pigmented/bluish-green leaves, reduction in leaf inclination (third to fifth leaves), shoot dry weight, root tips, MEC, convex hull, and increase in average root diameter and root dry weight were observed under P-deficient conditions (Anandan et al., 2021). Subsequently, hierarchical clustering (Supplementary Figure 4) sub-clustered the tolerant genotypes into three clades on the basis of geometric traits and root parameters. The contribution of greater values of geometric traits made tolerant genotypes (Dular, Kasalath, and IC459373) into a separate clade. Compared with the other genotypes, the relative expression of traits in Kasalath was least affected by deficient P, and this can be observed from Supplementary Figure 4, where it formed a separate clade. Besides, The Kasalath clade joined with the neighbouring clade have better-performing genotypes under deficient and control conditions, while the genotypes represented by greater total root length, root surface area, projected root area, and root tips under P-deficient conditions separated from the susceptible clade but grouped with the known tolerant clade. This hypothesis was confirmed by PCA further (Figure 3). The genotype with high biomass (shoot or root) exhibited a major trait that distinguishes it from the other genotypes. Genotypes with the greater geometric value used to have more shoot biomass with larger root systems were categorised as low P-tolerant, while genotypes with poor biomass and high tissue P were regarded as a poor performer under low P conditions. This signifies the importance of biomass in differentiating genotypes in a low P environment. The varying tolerant traits (geometric or root biomass) between groups of the genotypes may suggest the possibility of adopting different tolerant mechanisms to maintain their growth at the seedling stage under low P conditions. Irrespective of genotypes and treatments, P content was more in root than the shoot. Besides, the concentration of P was found to be lower in shoots and roots of all highly tolerant genotypes (Dular, Kasalath, and IC459373) compared with moderately tolerant or susceptible genotypes. The increased shoot biomass with reduced root numbers (Figure 3; Supplementary Figure 4), total root length, projected root area and surface root area, and root tips were observed intolerant than moderately tolerant genotypes. Reducing few numbers of lateral/crown roots and root tips might have diverted metabolites/photosynthates and P for increasing shoot biomass under P-deficient conditions. This was very well-supported by the negative correlation between root diameter and the number of root tips. An increase in root circumference (diameter) with maximum root length might have increased dry root biomass. Therefore, dry biomass should be used as a scale to classify genotypes, and this has been widely acknowledged (Dissanayaka et al., 2018). The increase in maximum root length and root thickness is directly proportional to the increase in root volume. The tolerant genotypes were observed to have higher volumes under P-deficient conditions.

Measuring economically relevant trait grain yield is not possible in all experiments, specifically at the seedling stage or handling large segregating generations where destructive sampling to study biomass is not viable. In a low P environment, shoot biomass, leaf number, and area are the most affected traits (Neilson et al., 2015), as most photosynthates are translated into root biomass in susceptible and moderately tolerant genotypes. Therefore, an imaging technique that measures MEC and convex hull based on leaf number, area, and inclination is observed to be an important trait contributing to plant biomass. The methodology (imaging technique) used here could provide insights into the mechanism by which low P affects leaf growth. The heat map generated combining traits, and genotypes of both the treatments were quite informative. They grouped the genotypes behaving in a similar manner across treatments. This supports the outcome of classification of traits *via* Ward's D distance where MEC, convex hull, WPA, and TVA form separate clusters indicated their significance among the traits under P-deficient conditions. Hence, these geometric traits may be considered as selection criteria for identifying low P-tolerant rice genotypes. The above highlighted geometric and root traits were explored to identify tolerant genotypes using stress-tolerant indices such as MPI, MRP, REI, TOL, STI, SSI, and DTE. Among them, MPI, MRP, and REI were found to be useful indices having r of >0.91 for the following traits: MEC, convex hull, calliper length, SPAD, shoot length, stem dry weight, fourth and fifth leaf weights, average root diameter, and shoot and root dry weight. The tolerance indices of MPI, MRP, and REI, and 11 mentioned morphological and geometric traits (Supplementary Table 5) helped to clearly differentiate low P-tolerant (Kasalath, IC459373, Dular, Sidhanta, Shankar, Meher, and Tanmayee) and susceptible genotypes (Suphala, Sarathi, Pratikshya, and Subhadra). The output of the above-mentioned indices were similar to the output of the PCA analysis (Figure 3). We switched from the destructive to the non-destructive way of evaluating genotypes by adapting geometric traits (MEC, convex hull, and calliper length), as surrogate traits would yield promising results in identifying low P-tolerant genotypes at the seedling stage. The methodology and traits identified with non-destructive imaging techniques in this experiment would be highly valuable for plant physiologists and breeders involved in identifying and developing rice genotypes for low P environments.

## Conclusion

This study explains the importance and utilisation of image-based phenotyping to select geometric traits, and identifies tolerant rice genotypes under low P conditions. The geometric traits such as MEC and convex hull highlighted their supremacy over the others in differentiating various rice genotypes, which is quite evident from various statistical analyses. In addition, shoot length, stem and root biomass, total root length, root volume, and root surface area could be further utilised as surrogate traits in the absence of an imaging technique at the seedling stage of the crop. Leaves positioned from third to fifth played a crucial role in P mobilisation to different plant parts; thus, maintaining plant architecture under P-deficient conditions to stabilise final yield. In addition, leaf angle, a critical yield determining factor, is reduced in the low P environment. Thus, the decline in leaf biomass and changes in the photosynthetic process create the scope for further determination of the underlying mechanism behind it. Concurrently, these geometric traits and other morphological and physiological traits were further utilised to identify the tolerant genotypes based on several stress indices. MPI, MRP, and REI displayed better responses for several traits establishing their usefulness under low P conditions. Overall, this study not only emphasises the importance of image-based phenotyping, which screens genotypes at the seedling stage, but also highlights the role of geometric traits in determining the final yield. Still, further research is needed to study the variation at the transcriptome and proteome levels, and the genes responsible for modification in underground traits accompanying alteration in photosynthetic processes and exudation of organic acids responsible for P mobilisation under P-deprived conditions.

## Data Availability Statement

The original contributions presented in the study are included in the article/Supplementary Material, further inquiries can be directed to the corresponding author/s.

## Author Contributions

AA, RP, and BB designed the experiments. BB and NP executed the experiments and performed data collection. AA performed data analysis and visualisation. BB and AA interpreted the data. BB, AA, AM, RP, BP, and JA wrote and critically revised the manuscript. All authors read and approved the final manuscript.

## Conflict of Interest

The authors declare that the research was conducted in the absence of any commercial or financial relationships that could be construed as a potential conflict of interest.

## Publisher's Note

All claims expressed in this article are solely those of the authors and do not necessarily represent those of their affiliated organizations, or those of the publisher, the editors and the reviewers. Any product that may be evaluated in this article, or claim that may be made by its manufacturer, is not guaranteed or endorsed by the publisher.

## Supplementary Material

Supplementary Figure 1 PCA biplot of 68 genotypes based on the variance in eight morpho-physiological traits measured in P stress environment, explained by two axes [PC1 and PC2, selected genotypes (orange) and non-selected genotypes (green)].

Supplementary Figure 2 Hierarchical clustering of the genotypes based on the level of expression of morpho-physiological traits in low P environment.

Supplementary Figure 3 Hierarchical clustering of the 38 morpho-physiological and geometric traits based on Ward's method.

Supplementary Figure 4 Hierarchical clustering and heat map of the 18 genotypes and 38 morpho-physiological and geometric traits. Each column represents a genotype, and each row represents a trait. The horizontal bar represents the treatment difference. Deficient (D) represented by pink colour, and Control (C) is represented by grey colour. The genotypes are grouped into two major clusters, and those having better shoot growth under deficient conditions and control are separated from the other genotypes.

Supplementary Table 1 List of genotypes used in this study.

Supplementary Table 2 Variability parameters of traits measured in rice genotypes raised under 0.5ppm of phosphorus.

Supplementary Table 3 Variability parameters of traits measured in rice genotypes raised in hydrophonics with full phosphorous.

Supplementary Table 4 Percent variation for significant traits of rice genotypes under both P deficient and control condition.

Supplementary Table 5 Stress tolerance indices and significant traits measured under P deficient and control condition.
