# Supplementary figures and images for: Improvement of Phosphorus Use Efficiency in Rice by Adopting Image-Based Phenotyping and Tolerant Indices
Source: Front Plant Sci. 2021 Aug 31;12:717107. doi: 10.3389/fpls.2021.717107 (PMC8438534; doi:10.3389/fpls.2021.717107)

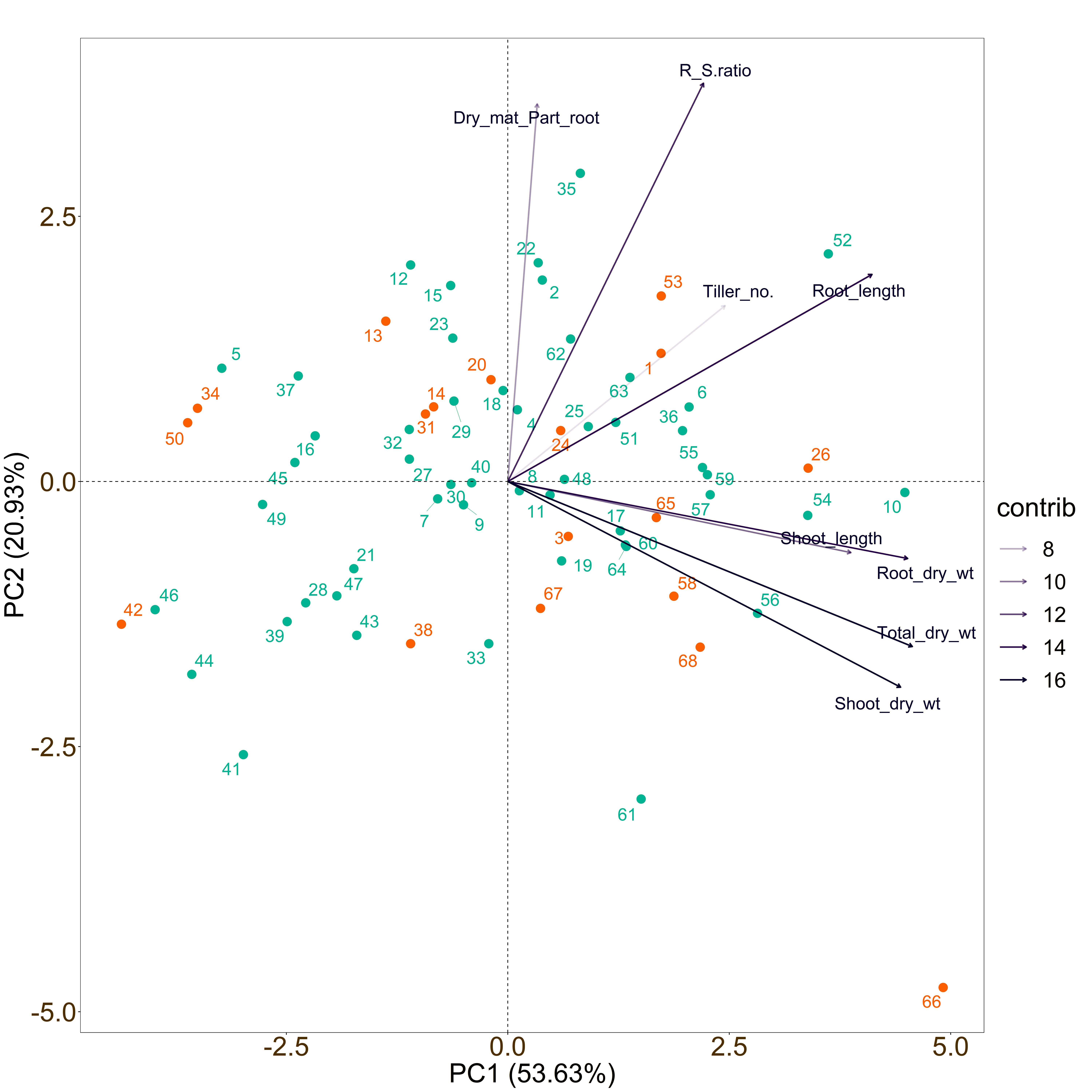

Supplement: Supplementary Figure 1 — PCA biplot of 68 genotypes based on the variance in eight morpho-physiological traits measured in P stress environment, explained by two axes [PC1 and PC2, selected genotypes (orange) and non-selected genotypes (green)]. [file Image_1.JPEG]

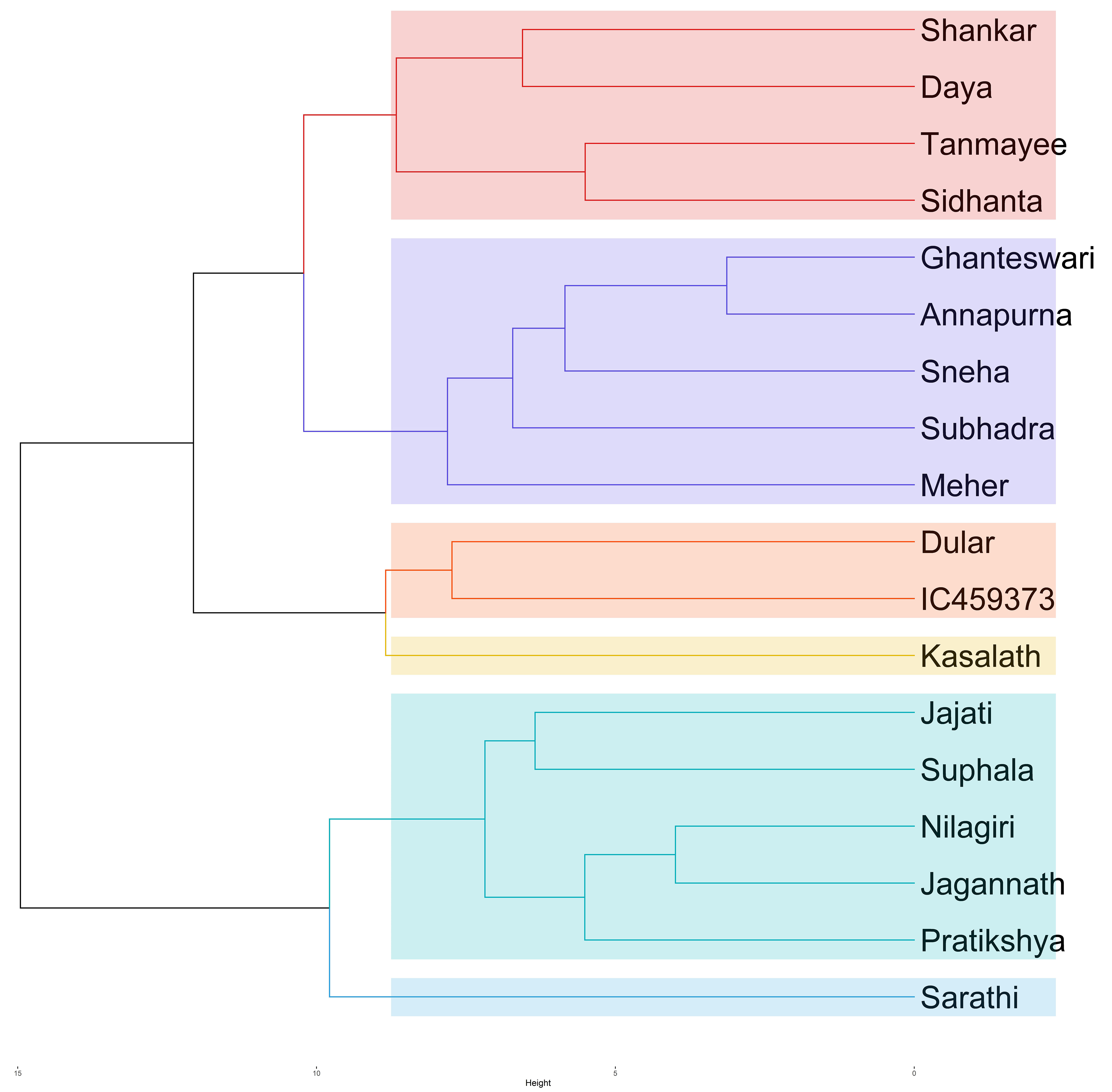

Supplement: Supplementary Figure 2 — Hierarchical clustering of the genotypes based on the level of expression of morpho-physiological traits in low P environment. [file Image_2.JPEG]

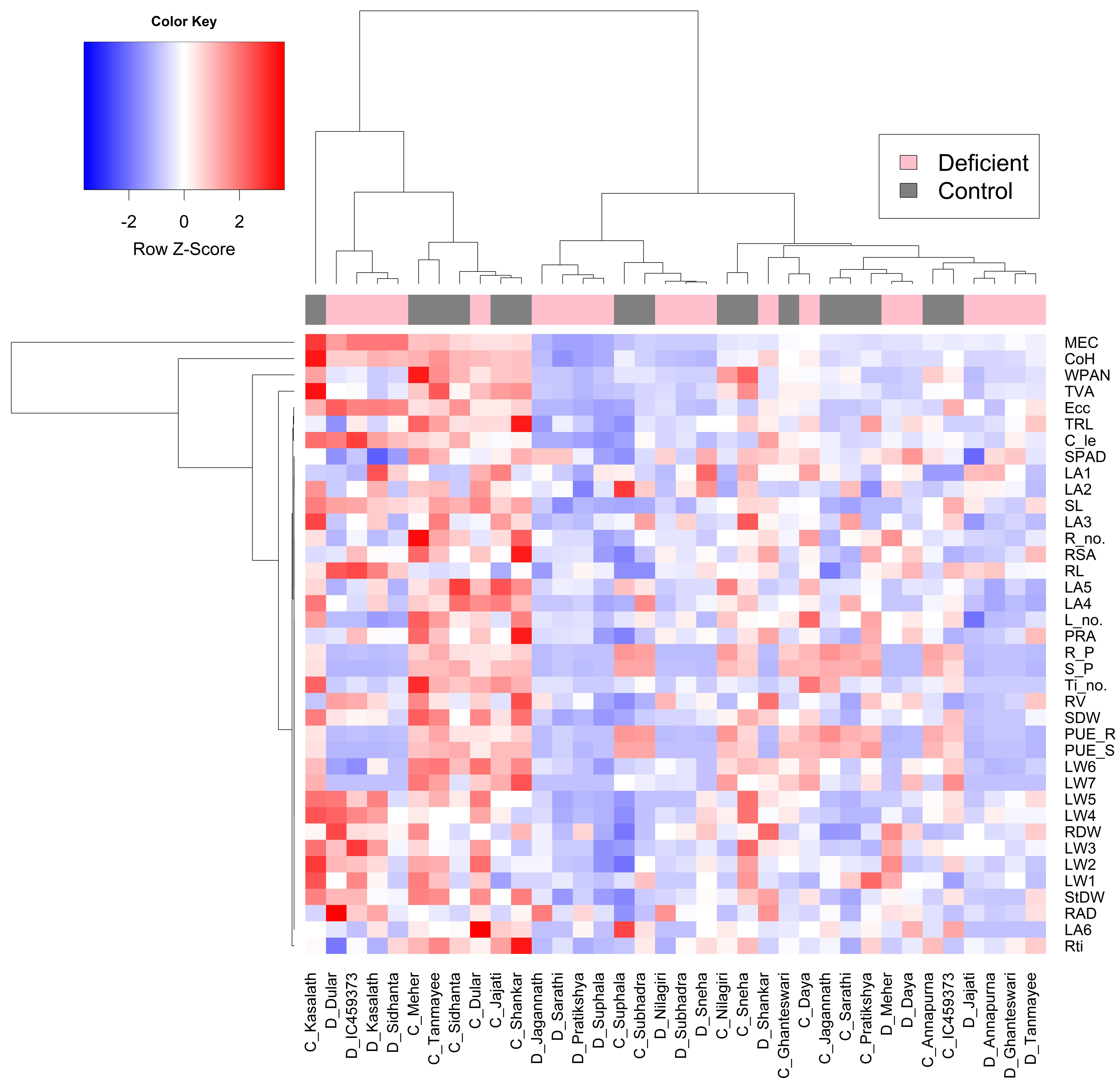

Supplement: Supplementary Figure 4 — Hierarchical clustering and heat map of the 18 genotypes and 38 morpho-physiological and geometric traits. Each column represents a genotype, and each row represents a trait. The horizontal bar represents the treatment difference. Deficient (D) represented by pink colour, and Control (C) is represented by grey colour. The genotypes are grouped into two major clusters, and those having better shoot growth under deficient conditions and control are separated from the other genotypes. [file Image_4.JPEG]
