# Supplementary material for: Improvement of Phosphorus Use Efficiency in Rice by Adopting Image-Based Phenotyping and Tolerant Indices
Source: Front Plant Sci. 2021 Aug 31;12:717107. doi: 10.3389/fpls.2021.717107 (PMC8438534; doi:10.3389/fpls.2021.717107)
Supplement: Supplementary Table 1 — List of genotypes used in this study. [file Data_Sheet_1.docx]

**Table S1** List of genotypes used in the present study

| **Sl. no.** | **Genotype** | **Landrace/ Improved genotype** | **Sl. no.** | **Genotype** | **Landrace/ Improved genotype** | **Sl. no.** | **Genotype** | **Landrace/ Improved genotype** | **Sl. no.** | **Genotype** | **Landrace/ Improved genotype** |
| --- | --- | --- | --- | --- | --- | --- | --- | --- | --- | --- | --- |
| 1 | Meher* | Improved | 18 | Sebati | Improved | 35 | Bhoi | Improved | 52 | Kharavela | Improved |
| 2 | Rajeswari | Improved | 19 | Vandana | Improved | 36 | Parijat | Improved | 53 | Shankar* | Improved |
| 3 | Subhadra* | Improved | 20 | Sneha* | Improved | 37 | Kalinga-3 | Improved | 54 | Keshari | Improved |
| 4 | Asutosh | Improved | 21 | Rambha | Improved | 38 | Sidhanta* | Improved | 55 | Anjali | Improved |
| 5 | Hiranmayi | Improved | 22 | Pooja | Improved | 39 | Indrabati | Improved | 56 | Gouri | Improved |
| 6 | Pratap | Improved | 23 | Mahanadi | Improved | 40 | Urbashi | Improved | 57 | Konark | Improved |
| 7 | Lalitgiri | Improved | 24 | Annapurna* | Improved | 41 | Jagabandhu | Improved | 58 | Nilagiri* | Improved |
| 8 | Mrunalini | Improved | 25 | Vanaprabha | Improved | 42 | Jajati* | Improved | 59 | Mahalaxmi | Improved |
| 9 | Pradip | Improved | 26 | Jagannath* | Improved | 43 | Kanchan | Improved | 60 | Heera | Improved |
| 10 | Pathara | Improved | 27 | Udayagiri | Improved | 44 | Tejaswini | Improved | 61 | Khandagiri | Improved |
| 11 | Pratibha | Improved | 28 | Samanta | Improved | 45 | Hasanta | Improved | 62 | Hema | Improved |
| 12 | Ranidhan | Improved | 29 | Birupa | Improved | 46 | Surendra | Improved | 63 | Manika | Improved |
| 13 | Suphala* | Improved | 30 | Rudra | Improved | 47 | Badami | Improved | 64 | Annada | Improved |
| 14 | Sarathi* | Improved | 31 | Ghanteswari* | Improved | 48 | Prachi | Improved | 65 | Tanmayee* | Improved |
| 15 | Manaswini | Improved | 32 | Bhuban | Improved | 49 | Gajapati | Improved | 66 | Kasalath* | Landrace |
| 16 | Uphara | Improved | 33 | Sabitree | Improved | 50 | Pratikshya* | Improved | 67 | IC459373* | Landrace |
| 17 | Ramachandi | Improved | 34 | Daya* | Improved | 51 | Bhanja | Improved | 68 | Dular* | Landrace |

* Selected 18 rice accessions used for hydroponic study
