# Supplementary material for: Improvement of Phosphorus Use Efficiency in Rice by Adopting Image-Based Phenotyping and Tolerant Indices
Source: Front Plant Sci. 2021 Aug 31;12:717107. doi: 10.3389/fpls.2021.717107 (PMC8438534; doi:10.3389/fpls.2021.717107)
Supplement: Supplementary Table 2 — Variability parameters of traits measured in rice genotypes raised under 0.5ppm of phosphorus. [file Data_Sheet_2.docx]

**Table S2. Variability parameters of traits measured in rice genotypes raised under 0.5ppm of phosphorus**

| **Variate** | **ECV** | **GCV** | **PCV** | **H²** | **Gen.Adv as % of Mean** | **Minimum** | **Maximum** | **Mean** | **95.0% Lower Confidence Limit** | **95.0% Upper Confidence Limit** | **SD** | **CV** | **Skewness** |
| --- | --- | --- | --- | --- | --- | --- | --- | --- | --- | --- | --- | --- | --- |
| Shoot length | 8.51 | 22.53 | 24.08 | 0.88 | 55.64 | 23.25 | 50.89 | 36.37 | 32.16 | 40.59 | 8.48 | 0.23 | 0.36 |
| Tiller number | 3.31 | 2.06 | 3.90 | 0.28 | 2.87 | 1.00 | 1.11 | 1.02 | 1.00 | 1.04 | 0.03 | 0.03 | 1.38 |
| Leaf number | 4.25 | 5.99 | 7.35 | 0.67 | 12.90 | 4.60 | 6.00 | 5.38 | 5.20 | 5.56 | 0.36 | 0.07 | -0.10 |
| Root number | 9.59 | 15.33 | 18.09 | 0.72 | 34.32 | 13.40 | 26.10 | 17.44 | 15.99 | 18.89 | 2.92 | 0.17 | 1.64 |
| Root length | 8.49 | 16.46 | 18.52 | 0.79 | 38.62 | 10.46 | 21.08 | 15.50 | 14.15 | 16.85 | 2.72 | 0.18 | 0.61 |
| SPAD | 7.02 | 9.22 | 11.59 | 0.63 | 19.38 | 25.56 | 35.89 | 31.28 | 29.65 | 32.91 | 3.28 | 0.11 | -0.42 |
| 1st leaf weight | 33.77 | 19.23 | 38.86 | 0.25 | 25.13 | 0.001 | 0.003 | 0.002 | 0.001 | 0.002 | 0.000 | 0.31 | 1.79 |
| 2nd leaf weight | 21.82 | 16.21 | 27.18 | 0.36 | 25.51 | 0.002 | 0.006 | 0.004 | 0.003 | 0.004 | 0.001 | 0.22 | 0.88 |
| 3rd leaf weight | 16.75 | 24.86 | 29.98 | 0.69 | 54.43 | 0.004 | 0.013 | 0.008 | 0.007 | 0.009 | 0.002 | 0.28 | 1.33 |
| 4th leaf weight | 12.74 | 29.09 | 31.76 | 0.84 | 70.35 | 0.009 | 0.022 | 0.013 | 0.011 | 0.015 | 0.004 | 0.31 | 1.17 |
| 5th leaf weight | 24.87 | 35.38 | 43.25 | 0.67 | 76.42 | 0.009 | 0.032 | 0.017 | 0.014 | 0.021 | 0.007 | 0.40 | 1.05 |
| Stem dry weight | 9.94 | 33.99 | 35.41 | 0.92 | 86.12 | 0.014 | 0.051 | 0.031 | 0.025 | 0.036 | 0.011 | 0.35 | 0.43 |
| Shoot weight | 14.91 | 26.89 | 30.75 | 0.77 | 62.09 | 0.043 | 0.133 | 0.085 | 0.073 | 0.097 | 0.025 | 0.29 | 0.33 |
| Root dry weight | 9.93 | 30.47 | 32.05 | 0.90 | 76.47 | 0.010 | 0.034 | 0.020 | 0.017 | 0.023 | 0.006 | 0.31 | 0.61 |
| Whole plant area | 23.27 | 18.45 | 29.70 | 0.39 | 30.27 | 2957.75 | 7923.67 | 5270.32 | 4622.34 | 5918.29 | 1303.02 | 0.25 | 0.19 |
| Top view area | 41.39 | 9.86 | 42.54 | 0.05 | 6.03 | 1094.96 | 3612.94 | 2288.18 | 1936.80 | 2639.55 | 706.59 | 0.31 | 0.26 |
| Shoot P | 15.06 | 32.06 | 35.42 | 0.82 | 76.59 | 0.36 | 1.12 | 0.58 | 0.49 | 0.68 | 0.20 | 0.34 | 1.10 |
| Root P | 25.99 | 27.27 | 37.67 | 0.52 | 52.11 | 0.34 | 1.16 | 0.58 | 0.48 | 0.67 | 0.19 | 0.33 | 1.78 |
| Convex hull | 27.90 | 45.70 | 53.54 | 0.73 | 102.96 | 8093.25 | 45768.42 | 24393.75 | 18356.20 | 30431.31 | 12140.95 | 0.50 | 0.68 |
| Calliper length | 19.57 | 22.90 | 30.12 | 0.58 | 45.97 | 219.84 | 530.55 | 329.06 | 285.28 | 372.85 | 88.05 | 0.27 | 0.91 |
| Eccentricity | 21.02 | 26.39 | 33.74 | 0.61 | 54.48 | 140.24 | 338.10 | 211.90 | 179.99 | 243.82 | 64.18 | 0.30 | 0.87 |
| Mini enclosing circle | 28.38 | 51.60 | 58.89 | 0.77 | 119.37 | 28873.91 | 138890.16 | 68593.62 | 49708.62 | 87478.63 | 37975.98 | 0.55 | 1.08 |
| 1st Leaf angle | 53.62 | 5.67 | 53.92 | 0.01 | 1.57 | 10.13 | 50.89 | 32.70 | 26.47 | 38.93 | 12.54 | 0.38 | 0.08 |
| 2nd Leaf angle | 32.78 | 19.17 | 37.97 | 0.26 | 25.55 | 15.04 | 59.79 | 34.97 | 29.74 | 40.20 | 10.52 | 0.30 | 0.51 |
| 3rd Leaf angle | 39.30 | 22.35 | 45.21 | 0.24 | 29.17 | 13.01 | 52.52 | 26.69 | 21.96 | 31.43 | 9.52 | 0.36 | 1.34 |
| 4th Leaf angle | 48.80 | 27.09 | 55.82 | 0.24 | 34.71 | 6.25 | 29.31 | 13.14 | 10.28 | 16.01 | 5.77 | 0.44 | 1.40 |
| 5th Leaf angle | 91.80 | 21.00 | 89.36 | -0.06 | -13.02 | 4.69 | 18.48 | 9.37 | 6.63 | 12.10 | 3.82 | 0.41 | 1.54 |
| Total root Length | 18.97 | 12.59 | 22.76 | 0.31 | 18.37 | 81.72 | 162.98 | 122.53 | 111.32 | 133.73 | 22.54 | 0.18 | -0.19 |
| Proj. root Area | 19.35 | 14.62 | 24.25 | 0.36 | 23.28 | 3.31 | 7.32 | 5.32 | 4.79 | 5.85 | 1.07 | 0.20 | 0.32 |
| Root surf. Area | 19.35 | 14.62 | 24.26 | 0.36 | 23.27 | 10.38 | 23.00 | 16.71 | 15.05 | 18.37 | 3.35 | 0.20 | 0.32 |
| Average root diameter | 13.67 | 15.66 | 20.78 | 0.57 | 31.15 | 0.35 | 0.67 | 0.44 | 0.40 | 0.48 | 0.08 | 0.18 | 1.25 |
| Root Volume | 24.97 | 23.08 | 34.00 | 0.46 | 41.36 | 0.10 | 0.30 | 0.19 | 0.16 | 0.22 | 0.06 | 0.29 | 0.17 |
| Root Tips | 21.90 | 22.98 | 31.74 | 0.52 | 43.93 | 211.67 | 703.00 | 483.52 | 416.89 | 550.15 | 133.98 | 0.28 | -0.01 |
| PUE_S | 15.23 | 32.19 | 35.61 | 0.82 | 76.81 | 0.002 | 0.006 | 0.003 | 0.002 | 0.003 | 0.001 | 0.34 | 1.10 |
| PUE_R | 25.56 | 29.23 | 38.83 | 0.57 | 58.07 | 0.002 | 0.006 | 0.003 | 0.002 | 0.003 | 0.001 | 0.34 | 1.72 |

Note: GCV – Genotypic Coefficient of Variation, PCV – Phenotypic Coefficient of Variation, ECV – Environmental Coefficient of Variation, CV - Coefficient of Variation, SD - Standard Deviation, H²– heritability (Broad Sense).
