# Supplementary material for: Improvement of Phosphorus Use Efficiency in Rice by Adopting Image-Based Phenotyping and Tolerant Indices
Source: Front Plant Sci. 2021 Aug 31;12:717107. doi: 10.3389/fpls.2021.717107 (PMC8438534; doi:10.3389/fpls.2021.717107)
Supplement: Supplementary Table 3 — Variability parameters of traits measured in rice genotypes raised in hydrophonics with full phosphorous. [file Data_Sheet_3.docx]

**Table S3.** Variability parameters of traits measured in rice genotypes raised in hydrophonics with full phosphorous

| **Variate** | **ECV** | **GCV** | **PCV** | **H²** | **Gen.Adv as % of Mean** | **Minimum** | **Maximum** | **Mean** | **95.0% Lower Confidence Limit** | **95.0% Upper Confidence Limit** | **SD** | **CV** | **Skewness** |
| --- | --- | --- | --- | --- | --- | --- | --- | --- | --- | --- | --- | --- | --- |
| Shoot length | 5.93 | 24.18 | 24.89 | 0.94 | 61.99 | 26.05 | 55.87 | 40.78 | 35.81 | 45.76 | 10.01 | 0.25 | -0.16 |
| Tiller number | 25.81 | 9.42 | 24.03 | -0.15 | -9.75 | 1.05 | 1.70 | 1.27 | 1.17 | 1.36 | 0.20 | 0.16 | 0.61 |
| Leaf number | 4.96 | 7.69 | 9.15 | 0.71 | 17.07 | 5.50 | 7.15 | 6.19 | 5.93 | 6.45 | 0.52 | 0.09 | 0.48 |
| Root number | 8.84 | 23.27 | 24.90 | 0.87 | 57.44 | 14.50 | 34.70 | 21.04 | 18.52 | 23.56 | 5.07 | 0.24 | 1.30 |
| Root length | 9.83 | 14.21 | 17.28 | 0.68 | 30.84 | 9.25 | 16.80 | 13.00 | 11.98 | 14.02 | 2.06 | 0.16 | -0.32 |
| SPAD | 3.87 | 5.83 | 7.00 | 0.69 | 12.83 | 27.40 | 36.20 | 32.20 | 31.17 | 33.24 | 2.08 | 0.06 | -0.18 |
| 1st leaf weight | 46.02 | 27.71 | 53.72 | 0.27 | 37.73 | 0.001 | 0.003 | 0.002 | 0.001 | 0.002 | 0.001 | 0.43 | 0.47 |
| 2nd leaf weight | 19.74 | 23.57 | 30.74 | 0.59 | 47.70 | 0.002 | 0.007 | 0.004 | 0.004 | 0.005 | 0.001 | 0.27 | 0.62 |
| 3rd leaf weight | 18.58 | 24.99 | 31.14 | 0.64 | 52.95 | 0.005 | 0.012 | 0.008 | 0.007 | 0.009 | 0.002 | 0.28 | 0.99 |
| 4th leaf weight | 15.73 | 30.69 | 34.49 | 0.79 | 72.11 | 0.007 | 0.024 | 0.014 | 0.011 | 0.016 | 0.004 | 0.33 | 0.78 |
| 5th leaf weight | 14.08 | 37.06 | 39.64 | 0.87 | 91.45 | 0.008 | 0.033 | 0.020 | 0.016 | 0.023 | 0.008 | 0.38 | 0.46 |
| 6th leaf weight | 20.11 | 35.26 | 40.59 | 0.76 | 80.86 | 0.008 | 0.033 | 0.021 | 0.017 | 0.025 | 0.008 | 0.38 | -0.08 |
| Stem dry weight | 15.19 | 41.14 | 43.85 | 0.88 | 101.89 | 0.010 | 0.058 | 0.036 | 0.028 | 0.043 | 0.015 | 0.43 | 0.19 |
| Shoot weight | 18.30 | 35.08 | 39.57 | 0.79 | 82.12 | 0.051 | 0.199 | 0.123 | 0.100 | 0.146 | 0.046 | 0.37 | 0.11 |
| Root dry weight | 16.12 | 34.54 | 38.11 | 0.82 | 82.62 | 0.005 | 0.028 | 0.015 | 0.012 | 0.017 | 0.005 | 0.36 | 0.61 |
| Whole plant area | 55.77 | 26.60 | 61.78 | 0.19 | 30.22 | 4235.14 | 31318.88 | 14354.24 | 10959.09 | 17749.38 | 6827.32 | 0.48 | 0.85 |
| Top view area | 50.57 | 38.17 | 63.35 | 0.36 | 60.70 | 1674.38 | 11939.59 | 5173.41 | 3827.95 | 6518.86 | 2705.58 | 0.52 | 1.04 |
| Shoot P | 4.24 | 12.45 | 13.15 | 0.90 | 31.11 | 5.42 | 8.82 | 7.54 | 7.06 | 8.02 | 0.97 | 0.13 | -0.91 |
| Root P | 11.53 | 18.36 | 21.68 | 0.72 | 41.04 | 5.90 | 11.25 | 8.56 | 7.70 | 9.41 | 1.72 | 0.20 | -0.03 |
| Convex hull | 35.04 | 32.03 | 47.47 | 0.46 | 57.05 | 20217.62 | 76374.49 | 35108.53 | 28039.16 | 42177.91 | 14215.85 | 0.41 | 1.51 |
| Calliper length | 17.16 | 11.06 | 20.42 | 0.29 | 15.82 | 235.61 | 483.58 | 335.44 | 308.06 | 362.83 | 55.07 | 0.16 | 0.96 |
| Eccentricity | 17.87 | 16.78 | 24.51 | 0.47 | 30.32 | 144.49 | 300.10 | 214.54 | 192.14 | 236.95 | 45.06 | 0.21 | 0.33 |
| Mini enclosing circle | 31.25 | 30.62 | 43.75 | 0.49 | 56.58 | 41972.14 | 167128.38 | 77371.32 | 62842.70 | 91899.94 | 29215.69 | 0.38 | 1.78 |
| 1st Leaf angle | 13.86 | 25.43 | 28.96 | 0.77 | 58.96 | 18.64 | 45.71 | 29.59 | 25.58 | 33.60 | 8.07 | 0.27 | 0.62 |
| 2nd Leaf angle | 16.64 | 26.09 | 30.94 | 0.71 | 58.06 | 19.89 | 59.70 | 36.17 | 31.02 | 41.31 | 10.35 | 0.29 | 0.48 |
| 3rd Leaf angle | 35.02 | 26.12 | 43.69 | 0.36 | 41.24 | 19.44 | 68.58 | 39.91 | 32.77 | 47.06 | 14.37 | 0.36 | 0.66 |
| 4th Leaf angle | 36.26 | 26.03 | 44.64 | 0.34 | 40.07 | 8.01 | 45.55 | 29.49 | 24.13 | 34.85 | 10.78 | 0.37 | -0.12 |
| 5th Leaf angle | 47.08 | 29.23 | 55.41 | 0.28 | 40.71 | 0.00 | 36.31 | 18.21 | 13.60 | 22.82 | 9.27 | 0.51 | 0.27 |
| 6th Leaf angle | 83.28 | 31.51 | 89.04 | 0.13 | 29.44 | 0.00 | 38.55 | 10.70 | 6.57 | 14.83 | 8.31 | 0.78 | 2.24 |
| Total root Length | 15.71 | 24.15 | 28.81 | 0.70 | 53.45 | 83.08 | 244.06 | 147.38 | 127.90 | 166.86 | 39.18 | 0.27 | 0.83 |
| Proj. root Area | 18.72 | 29.93 | 35.30 | 0.72 | 66.98 | 2.87 | 10.17 | 5.56 | 4.65 | 6.46 | 1.82 | 0.33 | 1.07 |
| Root surf. area | 18.71 | 29.93 | 35.30 | 0.72 | 66.98 | 9.00 | 31.96 | 17.46 | 14.62 | 20.30 | 5.71 | 0.33 | 1.07 |
| Average root diameter | 11.44 | 4.46 | 12.28 | 0.13 | 4.28 | 0.29 | 0.43 | 0.37 | 0.35 | 0.39 | 0.03 | 0.09 | -0.09 |
| Root Volume | 24.60 | 35.38 | 43.09 | 0.67 | 76.68 | 0.08 | 0.34 | 0.17 | 0.13 | 0.20 | 0.07 | 0.39 | 1.16 |
| Tips | 22.56 | 25.61 | 34.13 | 0.56 | 50.72 | 348.67 | 1176.83 | 648.56 | 551.25 | 745.86 | 195.67 | 0.30 | 0.95 |
| PUE_S | 4.73 | 12.26 | 13.14 | 0.87 | 30.19 | 0.017 | 0.028 | 0.023 | 0.022 | 0.025 | 0.003 | 0.13 | -0.72 |
| PUE_R | 12.34 | 18.89 | 22.57 | 0.70 | 41.76 | 0.018 | 0.036 | 0.027 | 0.024 | 0.029 | 0.006 | 0.21 | 0.05 |

Note: GCV – Genotypic Coefficient of Variation, PCV – Phenotypic Coefficient of Variation, ECV – Environmental Coefficient of Variation, CV - Coefficient of Variation, SD - Standard Deviation, H²– heritability (Broad Sense).
