# Supplementary material for: Improvement of Phosphorus Use Efficiency in Rice by Adopting Image-Based Phenotyping and Tolerant Indices
Source: Front Plant Sci. 2021 Aug 31;12:717107. doi: 10.3389/fpls.2021.717107 (PMC8438534; doi:10.3389/fpls.2021.717107)
Supplement: Supplementary Table 4 — Percent variation for significant traits of rice genotypes under both P deficient and control condition. [file Data_Sheet_4.docx]

**Table S4. Percent variation for significant traits of rice genotypes under both P deficient and control condition**

| **Traits** | **Meher** | **Subhadra** | **Suphala** | **Sarathi** | **Sneha** | **Annapurna** | **Jagannath** | **Ghanteswari** | **Daya** | **Sidhanta** | **Jajati** | **Pratikshya** | **Shankar** | **Nilagiri** | **Tanmayee** | **Kasalath** | **IC459373** | **Dular** |
| --- | --- | --- | --- | --- | --- | --- | --- | --- | --- | --- | --- | --- | --- | --- | --- | --- | --- | --- |
| **Shoot length (cm)** | -44.20 | 0.40 | 1.04 | -12.06 | -23.82 | -14.14 | 10.80 | -19.00 | -16.89 | -10.24 | -9.10 | -2.64 | -15.10 | -19.13 | -17.45 | -22.03 | 1.21 | -6.84 |
| **Leaf number** | -24.35 | -0.37 | -5.71 | -5.08 | -12.50 | -11.54 | 0.45 | -14.29 | -18.33 | -20.00 | -19.57 | -19.09 | -18.10 | -15.13 | -20.91 | -34.34 | -17.94 | -18.27 |
| **Root number** | -32.95 | -6.29 | -8.21 | -17.44 | -27.04 | -5.92 | 22.67 | -19.48 | -30.15 | -36.64 | -32.11 | -23.72 | -34.63 | -10.73 | -40.73 | -43.92 | 1.42 | -20.34 |
| **Root length (cm)** | 14.41 | 26.26 | 27.36 | 10.75 | 10.16 | 23.72 | 11.50 | 9.68 | 14.73 | 18.82 | 30.39 | 0.10 | 6.04 | -5.23 | 7.59 | 19.89 | 20.30 | 31.04 |
| **SPAD** | -7.77 | -1.65 | 2.25 | 9.99 | 2.39 | 0.42 | 8.59 | 3.39 | 6.27 | -13.00 | -25.60 | -4.56 | -3.02 | 8.97 | -12.63 | -24.45 | -5.08 | -13.01 |
| **3rd leaf weight (g)** | 11.26 | 1.90 | -5.94 | -3.57 | -75.02 | -9.93 | 13.71 | -23.84 | -2.78 | 2.17 | -0.67 | 10.00 | 30.72 | 11.00 | -0.93 | -12.26 | 43.75 | -10.47 |
| **4th leaf weight (g)** | -16.40 | 6.37 | 25.14 | -6.33 | -37.66 | 4.20 | 8.98 | -16.56 | -10.86 | -0.37 | -31.60 | 2.21 | 19.93 | -27.29 | -7.11 | -24.79 | 23.92 | 17.10 |
| **5th leaf weight (g)** | -78.23 | -2.39 | 22.73 | -13.83 | -58.11 | -23.31 | 18.16 | -10.79 | -17.25 | -41.07 | -47.91 | -18.35 | 14.95 | -23.35 | -18.82 | -5.24 | 9.80 | 0.69 |
| **6th leaf weight (g)** | -374.74 | 11.36 | -400.95 | 18.46 | -242.68 | -169.37 | -26.75 | -190.22 | -34.45 | -245.93 | -234.82 | -145.69 | -41.22 | -44.93 | -270.43 | -42.33 | 5.61 | -1215.56 |
| **Stem dry weight (g)** | -60.34 | 18.75 | 28.13 | -15.44 | -26.40 | -6.97 | 24.69 | -16.33 | -24.78 | -6.57 | -42.50 | -13.03 | -13.05 | -18.64 | -35.14 | -76.71 | 16.80 | -16.22 |
| **Shoot weight (g)** | -133.03 | 5.55 | -17.40 | -12.23 | -65.72 | -23.94 | 2.26 | -52.33 | -22.90 | -37.87 | -86.52 | -54.82 | -39.83 | -45.65 | -82.31 | -55.26 | -24.31 | -44.26 |
| **Root dry weight (g)** | -0.55 | 38.65 | 43.30 | 22.48 | 18.76 | 20.22 | 49.68 | 12.63 | 25.43 | 22.95 | 22.41 | 29.08 | 21.77 | 9.94 | 19.22 | 6.36 | 41.33 | 49.55 |
| **WPA (mm^2^)** | -604.94 | -42.02 | -73.29 | -167.86 | -355.52 | -160.92 | -97.32 | -88.05 | -81.75 | -185.15 | -352.39 | -43.19 | -137.87 | -225.14 | -210.36 | -281.39 | -43.60 | -75.51 |
| **Shoot P (mg/g)** | -1130.9 | -1089.3 | -1109.5 | -630.2 | -924.1 | -932.0 | -1293.0 | -1011.1 | -1167.0 | -1561.8 | -1814.9 | -1891.7 | -1738.7 | -953.3 | -1758.6 | -1512.6 | -1411.9 | -1354.1 |
| **Root P (mg/g)** | -2157.3 | -1700.1 | -1481.5 | -754.2 | -1443.3 | -1205.9 | -2042.8 | -872.7 | -1719.0 | -990.3 | -1437.4 | -1313.4 | -1485.2 | -2064.9 | -1655.3 | -1366.1 | -1285.1 | -1387.6 |
| **Convex hull** | -120.09 | -26.43 | -110.46 | -184.33 | -76.42 | -7.07 | -36.46 | -34.66 | -53.02 | -1.94 | -94.45 | -134.81 | -6.66 | -53.81 | -99.46 | -66.87 | 25.83 | -7.12 |
| **Caliper length** | -19.96 | -10.93 | -7.17 | -18.20 | -6.91 | -11.49 | -28.35 | -1.41 | -8.89 | -2.63 | -7.53 | -26.28 | 20.42 | -0.53 | -17.88 | -12.67 | 36.07 | 25.93 |
| **Eccentricity** | -23.18 | -8.37 | -3.03 | -5.45 | -9.43 | -11.36 | -8.75 | -4.97 | -6.95 | 2.56 | -9.00 | -17.43 | -8.28 | -4.06 | -7.25 | 12.61 | 9.54 | 31.53 |
| **MEC** | -82.00 | -6.87 | -33.02 | -101.49 | -29.82 | -3.38 | -62.08 | -0.18 | -30.21 | 32.75 | -37.30 | -90.52 | -52.57 | -26.15 | -62.92 | -21.88 | 54.50 | 26.27 |
| **Root average diameter (mm)** | 5.78 | 14.90 | 20.86 | 9.49 | 8.76 | -3.63 | 26.05 | -3.26 | 15.37 | 6.21 | 6.34 | 11.78 | 19.27 | 55.77 | 13.75 | 39.07 | 35.74 | 55.18 |
| **Root volume (cm^3^)** | -47.51 | 27.91 | 20.14 | 29.62 | 4.11 | -18.91 | 26.13 | -5.01 | 25.45 | 0.42 | -29.60 | -30.98 | -12.75 | 49.29 | 14.92 | 37.02 | 59.22 | 13.59 |
| **Tips** | -43.90 | -6.57 | -6.94 | 38.72 | -11.33 | -16.57 | -28.93 | 13.49 | 3.72 | -2.25 | -12.28 | -50.29 | -63.04 | -40.01 | -20.22 | -10.79 | 32.08 | -65.81 |
| **PUE_S** | -650.32 | -623.15 | -636.54 | -318.26 | -513.62 | -518.58 | -758.54 | -571.58 | -674.97 | -937.88 | -1105.36 | -1155.99 | -1053.79 | -532.76 | -1068.57 | -904.27 | -838.04 | -798.98 |
| **PUE_R** | -1333.18 | -1028.36 | -883.40 | -400.36 | -857.77 | -700.26 | -1257.05 | -479.19 | -1040.77 | -557.59 | -854.91 | -772.13 | -886.66 | -1270.96 | -1604.28 | -733.19 | -752.97 | -821.22 |
