# Supplementary material for: Improvement of Phosphorus Use Efficiency in Rice by Adopting Image-Based Phenotyping and Tolerant Indices
Source: Front Plant Sci. 2021 Aug 31;12:717107. doi: 10.3389/fpls.2021.717107 (PMC8438534; doi:10.3389/fpls.2021.717107)
Supplement: Supplementary Table 5 — Stress tolerance indices and significant traits measured under P deficient and control condition. [file Data_Sheet_5.docx]

**Table S5. Stress tolerance indices and significant traits measured under P deficient and control condition**

| **Genotype** | **Trait** | **Stress-tolerant indices** | | | | | | |
| --- | --- | --- | --- | --- | --- | --- | --- | --- |
|  |  | **TOL** | **STI** | **SSI** | **DTE%** | **MPI** | **MRP** | **REI** |
| IC459373 | Minimum  Enclosing  circle | 75075.04 | 0.46 | 4.82 | 45.50 | 100225.22 | 2.75 | 1.72 |
| Kasalath |  | 30005.13 | 0.82 | 1.59 | 82.05 | 152125.82 | 4.27 | 4.56 |
| Dular |  | 31645.89 | 0.74 | 2.32 | 73.73 | 104645.10 | 2.93 | 2.13 |
| Tanmayee |  | 41228.65 | 0.61 | 3.42 | 61.38 | 86139.58 | 2.39 | 1.39 |
| Nilagiri |  | 12339.08 | 0.79 | 1.83 | 79.27 | 53358.71 | 1.50 | 0.56 |
| Shankar |  | 31435.78 | 0.66 | 3.05 | 65.54 | 75516.35 | 2.10 | 1.09 |
| Pratikshya |  | 26170.37 | 0.52 | 4.20 | 52.49 | 41995.84 | 1.16 | 0.32 |
| Jajati |  | 23860.78 | 0.73 | 2.40 | 72.84 | 75907.32 | 2.12 | 1.12 |
| Sidhanta |  | 45490.52 | 0.67 | 2.90 | 67.25 | 116144.90 | 3.24 | 2.58 |
| Daya |  | 16986.75 | 0.77 | 2.05 | 76.80 | 64719.00 | 1.81 | 0.82 |
| Ghanteswari |  | 126.63 | 1.00 | 0.02 | 99.82 | 69459.88 | 1.97 | 0.96 |
| Jagannath |  | 22688.53 | 0.62 | 3.39 | 61.70 | 47893.43 | 1.33 | 0.43 |
| Annapurna |  | 2102.07 | 0.97 | 0.29 | 96.73 | 63293.70 | 1.79 | 0.80 |
| Sneha |  | 13978.91 | 0.77 | 2.03 | 77.03 | 53873.19 | 1.51 | 0.57 |
| Sarathi |  | 29305.00 | 0.50 | 4.46 | 49.63 | 43526.41 | 1.20 | 0.33 |
| Suphala |  | 10420.02 | 0.75 | 2.20 | 75.17 | 36762.13 | 1.03 | 0.26 |
| Subhadra |  | 3215.52 | 0.94 | 0.57 | 93.57 | 48402.01 | 1.37 | 0.47 |
| Meher |  | 46346.73 | 0.55 | 3.99 | 54.95 | 79695.91 | 2.20 | 1.16 |
| IC459373 | Convex hull | 10424.56 | 0.74 | 0.85 | 74.17 | 35141.27 | 2.38 | 1.41 |
| Kasalath |  | 30606.07 | 0.60 | 1.31 | 59.93 | 61071.46 | 4.05 | 4.08 |
| Dular |  | 2910.79 | 0.93 | 0.22 | 93.35 | 42316.95 | 2.92 | 2.09 |
| Tanmayee |  | 25894.83 | 0.50 | 1.63 | 50.13 | 38982.06 | 2.55 | 1.58 |
| Nilagiri |  | 9006.65 | 0.65 | 1.15 | 65.02 | 21241.48 | 1.42 | 0.50 |
| Shankar |  | 2636.60 | 0.94 | 0.20 | 93.75 | 40895.16 | 2.82 | 1.95 |
| Pratikshya |  | 15106.07 | 0.43 | 1.88 | 42.59 | 18758.40 | 1.21 | 0.34 |
| Jajati |  | 20318.91 | 0.51 | 1.59 | 51.43 | 31672.74 | 2.07 | 1.05 |
| Sidhanta |  | 851.21 | 0.98 | 0.06 | 98.09 | 44238.30 | 3.07 | 2.28 |
| Daya |  | 11633.33 | 0.65 | 1.14 | 65.35 | 27759.29 | 1.86 | 0.86 |
| Ghanteswari |  | 7589.02 | 0.74 | 0.84 | 74.26 | 25689.27 | 1.74 | 0.75 |
| Jagannath |  | 5719.43 | 0.73 | 0.88 | 73.28 | 18548.68 | 1.25 | 0.39 |
| Annapurna |  | 1489.87 | 0.93 | 0.22 | 93.39 | 21808.95 | 1.51 | 0.55 |
| Sneha |  | 11750.17 | 0.57 | 1.42 | 56.68 | 21250.21 | 1.40 | 0.49 |
| Sarathi |  | 14918.53 | 0.35 | 2.13 | 35.17 | 15552.52 | 0.99 | 0.22 |
| Suphala |  | 13817.76 | 0.48 | 1.72 | 47.51 | 19418.04 | 1.26 | 0.38 |
| Subhadra |  | 4226.36 | 0.79 | 0.69 | 79.10 | 18104.44 | 1.23 | 0.38 |
| Meher |  | 24814.97 | 0.45 | 1.79 | 45.44 | 33071.34 | 2.14 | 1.10 |
| IC459373 | Caliper  length | 191.36 | 0.64 | 1.90 | 63.93 | 434.87 | 2.61 | 1.63 |
| Kasalath |  | 54.36 | 0.89 | 0.59 | 88.76 | 456.40 | 2.75 | 1.88 |
| Dular |  | 120.93 | 0.74 | 1.36 | 74.07 | 405.98 | 2.44 | 1.46 |
| Tanmayee |  | 55.63 | 0.85 | 0.80 | 84.84 | 339.02 | 2.04 | 1.03 |
| Nilagiri |  | 1.53 | 0.99 | 0.03 | 99.47 | 290.30 | 1.75 | 0.76 |
| Shankar |  | 88.21 | 0.80 | 1.07 | 79.58 | 387.83 | 2.33 | 1.35 |
| Pratikshya |  | 61.33 | 0.79 | 1.10 | 79.19 | 264.06 | 1.59 | 0.62 |
| Jajati |  | 22.56 | 0.93 | 0.37 | 93.00 | 310.96 | 1.87 | 0.87 |
| Sidhanta |  | 10.58 | 0.97 | 0.13 | 97.44 | 407.67 | 2.45 | 1.51 |
| Daya |  | 26.67 | 0.92 | 0.43 | 91.83 | 313.25 | 1.89 | 0.89 |
| Ghanteswari |  | 4.89 | 0.99 | 0.07 | 98.61 | 350.60 | 2.11 | 1.11 |
| Jagannath |  | 69.74 | 0.78 | 1.16 | 77.91 | 280.87 | 1.69 | 0.70 |
| Annapurna |  | 32.67 | 0.90 | 0.54 | 89.70 | 300.77 | 1.81 | 0.82 |
| Sneha |  | 18.04 | 0.94 | 0.34 | 93.54 | 270.21 | 1.63 | 0.66 |
| Sarathi |  | 44.51 | 0.85 | 0.81 | 84.60 | 266.78 | 1.60 | 0.64 |
| Suphala |  | 15.77 | 0.93 | 0.35 | 93.31 | 227.72 | 1.37 | 0.47 |
| Subhadra |  | 33.43 | 0.90 | 0.52 | 90.14 | 322.44 | 1.94 | 0.94 |
| Meher |  | 63.66 | 0.83 | 0.88 | 83.36 | 350.83 | 2.11 | 1.11 |
| IC459373 | SPAD | 1.50 | 0.95 | 1.61 | 95.16 | 30.27 | 1.91 | 0.91 |
| Kasalath |  | 6.25 | 0.80 | 6.55 | 80.35 | 28.69 | 1.81 | 0.81 |
| Dular |  | 3.58 | 0.88 | 3.84 | 88.49 | 29.31 | 1.85 | 0.85 |
| Tanmayee |  | 3.88 | 0.89 | 3.74 | 88.79 | 32.67 | 2.06 | 1.06 |
| Nilagiri |  | -3.01 | 1.10 | -3.28 | 109.85 | 32.06 | 2.02 | 1.02 |
| Shankar |  | 1.02 | 0.97 | 0.98 | 97.07 | 34.31 | 2.16 | 1.17 |
| Pratikshya |  | 1.42 | 0.96 | 1.45 | 95.64 | 31.84 | 2.01 | 1.01 |
| Jajati |  | 6.62 | 0.80 | 6.79 | 79.62 | 29.17 | 1.84 | 0.83 |
| Sidhanta |  | 3.62 | 0.88 | 3.83 | 88.50 | 29.66 | 1.87 | 0.87 |
| Daya |  | -2.25 | 1.07 | -2.23 | 106.69 | 34.77 | 2.19 | 1.20 |
| Ghanteswari |  | -1.15 | 1.04 | -1.17 | 103.50 | 33.40 | 2.11 | 1.11 |
| Jagannath |  | -2.92 | 1.09 | -3.13 | 109.40 | 32.54 | 2.05 | 1.05 |
| Annapurna |  | -0.14 | 1.00 | -0.14 | 100.42 | 33.13 | 2.09 | 1.09 |
| Sneha |  | -0.84 | 1.02 | -0.82 | 102.45 | 34.69 | 2.19 | 1.19 |
| Sarathi |  | -3.39 | 1.11 | -3.70 | 111.10 | 32.24 | 2.03 | 1.03 |
| Suphala |  | -0.63 | 1.02 | -0.77 | 102.30 | 27.72 | 1.75 | 0.76 |
| Subhadra |  | 0.49 | 0.98 | 0.54 | 98.38 | 30.02 | 1.89 | 0.89 |
| Meher |  | 2.61 | 0.93 | 2.40 | 92.79 | 34.90 | 2.20 | 1.21 |
| IC459373 | Shoot length | 1.39 | 0.97 | 0.22 | 97.33 | 51.25 | 2.65 | 1.75 |
| Kasalath |  | 10.09 | 0.82 | 1.50 | 81.95 | 50.82 | 2.61 | 1.70 |
| Dular |  | 3.48 | 0.94 | 0.53 | 93.60 | 52.63 | 2.72 | 1.84 |
| Tanmayee |  | 7.59 | 0.85 | 1.24 | 85.15 | 47.30 | 2.43 | 1.48 |
| Nilagiri |  | 6.21 | 0.84 | 1.34 | 83.94 | 35.57 | 1.83 | 0.84 |
| Shankar |  | 6.62 | 0.87 | 1.09 | 86.88 | 47.15 | 2.43 | 1.47 |
| Pratikshya |  | 0.76 | 0.97 | 0.21 | 97.43 | 29.16 | 1.51 | 0.57 |
| Jajati |  | 3.78 | 0.92 | 0.70 | 91.66 | 43.45 | 2.24 | 1.25 |
| Sidhanta |  | 4.55 | 0.91 | 0.77 | 90.72 | 46.73 | 2.41 | 1.45 |
| Daya |  | 5.62 | 0.86 | 1.20 | 85.55 | 36.05 | 1.86 | 0.86 |
| Ghanteswari |  | 5.99 | 0.84 | 1.33 | 84.03 | 34.52 | 1.78 | 0.79 |
| Jagannath |  | 1.62 | 0.95 | 0.41 | 95.10 | 32.15 | 1.66 | 0.69 |
| Annapurna |  | 4.77 | 0.88 | 1.03 | 87.61 | 36.07 | 1.86 | 0.86 |
| Sneha |  | 8.53 | 0.81 | 1.60 | 80.76 | 40.08 | 2.06 | 1.06 |
| Sarathi |  | 2.80 | 0.89 | 0.90 | 89.24 | 24.65 | 1.27 | 0.40 |
| Suphala |  | 0.72 | 0.97 | 0.22 | 97.40 | 27.35 | 1.41 | 0.50 |
| Subhadra |  | 0.89 | 0.97 | 0.26 | 96.89 | 28.20 | 1.46 | 0.53 |
| Meher |  | 12.96 | 0.69 | 2.55 | 69.35 | 35.79 | 1.83 | 0.83 |
| IC459373 | Stem dry  weight | 0.0079 | 0.83 | 1.12 | 83.20 | 0.043 | 2.47 | 1.53 |
| Kasalath |  | 0.0247 | 0.57 | 2.89 | 56.59 | 0.045 | 2.50 | 1.53 |
| Dular |  | 0.0076 | 0.86 | 0.93 | 86.04 | 0.051 | 2.92 | 2.13 |
| Tanmayee |  | 0.0143 | 0.74 | 1.73 | 74.00 | 0.048 | 2.73 | 1.87 |
| Nilagiri |  | 0.0047 | 0.84 | 1.05 | 84.29 | 0.027 | 1.57 | 0.62 |
| Shankar |  | 0.0067 | 0.88 | 0.77 | 88.46 | 0.055 | 3.16 | 2.48 |
| Pratikshya |  | 0.0032 | 0.88 | 0.77 | 88.47 | 0.026 | 1.51 | 0.57 |
| Jajati |  | 0.0097 | 0.70 | 1.99 | 70.17 | 0.028 | 1.58 | 0.62 |
| Sidhanta |  | 0.0020 | 0.94 | 0.41 | 93.84 | 0.031 | 1.83 | 0.82 |
| Daya |  | 0.0077 | 0.80 | 1.32 | 80.14 | 0.035 | 2.01 | 1.00 |
| Ghanteswari |  | 0.0037 | 0.86 | 0.94 | 85.96 | 0.024 | 1.40 | 0.48 |
| Jagannath |  | 0.0069 | 0.75 | 1.65 | 75.31 | 0.024 | 1.39 | 0.49 |
| Annapurna |  | 0.0016 | 0.93 | 0.43 | 93.49 | 0.023 | 1.34 | 0.44 |
| Sneha |  | 0.0092 | 0.79 | 1.39 | 79.11 | 0.039 | 2.26 | 1.28 |
| Sarathi |  | 0.0022 | 0.87 | 0.89 | 86.63 | 0.015 | 0.87 | 0.19 |
| Suphala |  | 0.0041 | 0.72 | 1.88 | 71.88 | 0.012 | 0.71 | 0.12 |
| Subhadra |  | 0.0053 | 0.81 | 1.25 | 81.25 | 0.026 | 1.48 | 0.55 |
| Meher |  | 0.0213 | 0.62 | 2.51 | 62.37 | 0.046 | 2.59 | 1.66 |
| IC459373 | 4th leaf  weight | -0.0049 | 1.31 | -9.88 | 131.43 | 0.018 | 2.73 | 1.82 |
| Kasalath |  | 0.0047 | 0.80 | 6.25 | 80.14 | 0.021 | 3.17 | 2.49 |
| Dular |  | -0.0038 | 1.21 | -6.49 | 120.63 | 0.021 | 3.08 | 2.35 |
| Tanmayee |  | 0.0009 | 0.93 | 2.09 | 93.36 | 0.013 | 1.96 | 0.96 |
| Nilagiri |  | 0.0031 | 0.79 | 6.74 | 78.56 | 0.013 | 1.92 | 0.91 |
| Shankar |  | -0.0026 | 1.25 | -7.83 | 124.89 | 0.012 | 1.76 | 0.76 |
| Pratikshya |  | -0.0002 | 1.02 | -0.71 | 102.26 | 0.009 | 1.38 | 0.48 |
| Jajati |  | 0.0034 | 0.76 | 7.55 | 75.99 | 0.013 | 1.88 | 0.87 |
| Sidhanta |  | 0.0001 | 1.00 | 0.12 | 99.63 | 0.013 | 2.02 | 1.02 |
| Daya |  | 0.0011 | 0.90 | 3.08 | 90.21 | 0.010 | 1.54 | 0.60 |
| Ghanteswari |  | 0.0021 | 0.86 | 4.47 | 85.80 | 0.014 | 2.02 | 1.02 |
| Jagannath |  | -0.0010 | 1.10 | -3.10 | 109.87 | 0.011 | 1.61 | 0.64 |
| Annapurna |  | -0.0006 | 1.04 | -1.38 | 104.38 | 0.014 | 2.10 | 1.10 |
| Sneha |  | 0.0060 | 0.73 | 8.60 | 72.64 | 0.019 | 2.81 | 1.93 |
| Sarathi |  | 0.0005 | 0.94 | 1.87 | 94.05 | 0.009 | 1.32 | 0.43 |
| Suphala |  | -0.0022 | 1.34 | -10.56 | 133.59 | 0.008 | 1.15 | 0.32 |
| Subhadra |  | -0.0007 | 1.07 | -2.14 | 106.80 | 0.010 | 1.51 | 0.57 |
| Meher |  | 0.0021 | 0.86 | 4.43 | 85.91 | 0.014 | 2.02 | 1.02 |
| IC459373 | 5th leaf  weight | 0.0024 | 0.90 | 0.79 | 90.20 | 0.023 | 2.29 | 1.31 |
| Kasalath |  | 0.0016 | 0.95 | 0.40 | 95.02 | 0.032 | 3.18 | 2.53 |
| Dular |  | 0.0002 | 0.99 | 0.06 | 99.31 | 0.032 | 3.21 | 2.57 |
| Tanmayee |  | 0.0042 | 0.84 | 1.28 | 84.16 | 0.024 | 2.45 | 1.49 |
| Nilagiri |  | 0.0028 | 0.81 | 1.53 | 81.07 | 0.013 | 1.35 | 0.45 |
| Shankar |  | 0.0033 | 0.85 | 1.21 | 85.05 | 0.020 | 2.03 | 1.02 |
| Pratikshya |  | 0.0020 | 0.84 | 1.25 | 84.50 | 0.012 | 1.21 | 0.37 |
| Jajati |  | 0.0060 | 0.68 | 2.61 | 67.61 | 0.016 | 1.55 | 0.58 |
| Sidhanta |  | 0.0061 | 0.71 | 2.35 | 70.89 | 0.018 | 1.78 | 0.77 |
| Daya |  | 0.0027 | 0.85 | 1.19 | 85.29 | 0.017 | 1.67 | 0.69 |
| Ghanteswari |  | 0.0019 | 0.90 | 0.79 | 90.26 | 0.019 | 1.88 | 0.88 |
| Jagannath |  | 0.0025 | 0.82 | 1.46 | 81.84 | 0.013 | 1.26 | 0.39 |
| Annapurna |  | 0.0036 | 0.81 | 1.52 | 81.10 | 0.017 | 1.74 | 0.75 |
| Sneha |  | 0.0121 | 0.63 | 2.96 | 63.25 | 0.027 | 2.68 | 1.70 |
| Sarathi |  | 0.0013 | 0.88 | 0.98 | 87.85 | 0.010 | 1.00 | 0.25 |
| Suphala |  | 0.0024 | 0.77 | 1.83 | 77.27 | 0.009 | 0.92 | 0.21 |
| Subhadra |  | 0.0003 | 0.98 | 0.19 | 97.66 | 0.012 | 1.21 | 0.37 |
| Meher |  | 0.0101 | 0.56 | 3.54 | 56.11 | 0.018 | 1.80 | 0.75 |
| IC459373 | Shoot dry  weight | 0.027 | 0.80 | 0.61 | 80.44 | 0.13 | 2.42 | 1.47 |
| Kasalath |  | 0.064 | 0.64 | 1.11 | 64.41 | 0.15 | 2.78 | 1.92 |
| Dular |  | 0.053 | 0.69 | 0.96 | 69.32 | 0.15 | 2.78 | 1.93 |
| Tanmayee |  | 0.079 | 0.55 | 1.41 | 54.85 | 0.14 | 2.52 | 1.55 |
| Nilagiri |  | 0.035 | 0.69 | 0.98 | 68.66 | 0.10 | 1.80 | 0.81 |
| Shankar |  | 0.053 | 0.72 | 0.89 | 71.52 | 0.16 | 3.02 | 2.28 |
| Pratikshya |  | 0.034 | 0.65 | 1.11 | 64.59 | 0.08 | 1.48 | 0.55 |
| Jajati |  | 0.057 | 0.54 | 1.45 | 53.61 | 0.09 | 1.75 | 0.74 |
| Sidhanta |  | 0.029 | 0.73 | 0.86 | 72.53 | 0.09 | 1.75 | 0.76 |
| Daya |  | 0.024 | 0.81 | 0.58 | 81.37 | 0.12 | 2.23 | 1.25 |
| Ghanteswari |  | 0.037 | 0.66 | 1.07 | 65.65 | 0.09 | 1.67 | 0.70 |
| Jagannath |  | 0.012 | 0.86 | 0.43 | 86.17 | 0.08 | 1.59 | 0.63 |
| Annapurna |  | 0.017 | 0.81 | 0.60 | 80.68 | 0.08 | 1.50 | 0.56 |
| Sneha |  | 0.060 | 0.60 | 1.24 | 60.34 | 0.12 | 2.27 | 1.28 |
| Sarathi |  | 0.006 | 0.89 | 0.34 | 89.11 | 0.06 | 1.06 | 0.28 |
| Suphala |  | 0.008 | 0.85 | 0.46 | 85.18 | 0.05 | 0.90 | 0.20 |
| Subhadra |  | 0.011 | 0.88 | 0.38 | 87.93 | 0.08 | 1.60 | 0.64 |
| Meher |  | 0.114 | 0.43 | 1.78 | 42.91 | 0.14 | 2.61 | 1.57 |
| IC459373 | Root dry  weight | -0.009 | 1.70 | 2.07 | 170.45 | 0.016 | 2.25 | 1.26 |
| Kasalath |  | -0.001 | 1.07 | 0.20 | 106.79 | 0.019 | 2.82 | 1.81 |
| Dular |  | -0.017 | 1.98 | 2.89 | 198.22 | 0.025 | 3.37 | 2.83 |
| Tanmayee |  | -0.004 | 1.24 | 0.70 | 123.80 | 0.020 | 2.86 | 1.93 |
| Nilagiri |  | -0.002 | 1.11 | 0.32 | 111.04 | 0.016 | 2.40 | 1.32 |
| Shankar |  | -0.007 | 1.28 | 0.82 | 127.82 | 0.027 | 3.92 | 3.65 |
| Pratikshya |  | -0.006 | 1.41 | 1.21 | 141.00 | 0.018 | 2.56 | 1.59 |
| Jajati |  | -0.004 | 1.29 | 0.85 | 128.89 | 0.015 | 2.22 | 1.17 |
| Sidhanta |  | -0.004 | 1.30 | 0.88 | 129.79 | 0.016 | 2.33 | 1.29 |
| Daya |  | -0.006 | 1.34 | 1.00 | 134.10 | 0.019 | 2.71 | 1.76 |
| Ghanteswari |  | -0.002 | 1.14 | 0.43 | 114.45 | 0.014 | 2.01 | 0.94 |
| Jagannath |  | -0.008 | 1.99 | 2.90 | 198.74 | 0.012 | 1.59 | 0.63 |
| Annapurna |  | -0.003 | 1.25 | 0.75 | 125.34 | 0.012 | 1.80 | 0.77 |
| Sneha |  | -0.004 | 1.23 | 0.68 | 123.10 | 0.021 | 2.97 | 2.08 |
| Sarathi |  | -0.002 | 1.29 | 0.85 | 129.01 | 0.010 | 1.41 | 0.48 |
| Suphala |  | -0.004 | 1.76 | 2.25 | 176.36 | 0.008 | 1.04 | 0.27 |
| Subhadra |  | -0.007 | 1.63 | 1.85 | 162.99 | 0.015 | 2.12 | 1.11 |
| Meher |  | 0.000 | 1.02 | 0.05 | 101.61 | 0.028 | 4.13 | 3.82 |
| IC459373 | Root  average  diameter | -0.121 | 1.36 | 1.79 | 135.74 | 0.40 | 1.95 | 0.95 |
| Kasalath |  | -0.141 | 1.39 | 1.95 | 139.07 | 0.43 | 2.11 | 1.11 |
| Dular |  | -0.237 | 1.55 | 2.76 | 155.18 | 0.55 | 2.67 | 1.75 |
| Tanmayee |  | -0.060 | 1.16 | 0.80 | 115.94 | 0.41 | 2.01 | 1.01 |
| Nilagiri |  | -0.188 | 1.56 | 2.79 | 155.77 | 0.43 | 2.10 | 1.08 |
| Shankar |  | -0.100 | 1.24 | 1.19 | 123.87 | 0.47 | 2.31 | 1.33 |
| Pratikshya |  | -0.053 | 1.13 | 0.67 | 113.35 | 0.42 | 2.10 | 1.10 |
| Jajati |  | -0.012 | 1.03 | 0.17 | 103.45 | 0.36 | 1.80 | 0.80 |
| Sidhanta |  | -0.016 | 1.04 | 0.22 | 104.40 | 0.37 | 1.85 | 0.85 |
| Daya |  | -0.066 | 1.18 | 0.91 | 118.16 | 0.40 | 1.95 | 0.95 |
| Ghanteswari |  | 0.012 | 0.97 | -0.16 | 96.74 | 0.36 | 1.81 | 0.81 |
| Jagannath |  | -0.140 | 1.35 | 1.76 | 135.22 | 0.47 | 2.29 | 1.30 |
| Annapurna |  | 0.013 | 0.96 | -0.18 | 96.37 | 0.36 | 1.79 | 0.80 |
| Sneha |  | -0.036 | 1.10 | 0.48 | 109.61 | 0.39 | 1.96 | 0.96 |
| Sarathi |  | -0.035 | 1.10 | 0.52 | 110.49 | 0.35 | 1.73 | 0.74 |
| Suphala |  | -0.077 | 1.26 | 1.32 | 126.35 | 0.33 | 1.64 | 0.67 |
| Subhadra |  | -0.060 | 1.18 | 0.88 | 117.51 | 0.37 | 1.84 | 0.84 |
| Meher |  | -0.024 | 1.06 | 0.29 | 105.78 | 0.42 | 2.11 | 1.11 |
